# Steroid nuclear receptor coactivator 2 controls immune tolerance by promoting induced T_reg_ differentiation via up-regulating Nr4a2

**DOI:** 10.1126/sciadv.abn7662

**Published:** 2022-06-15

**Authors:** Wencan Zhang, Xu Cao, Xiancai Zhong, Hongmin Wu, Mingye Feng, Yousang Gwack, Isakov Noah, Zuoming Sun

**Affiliations:** 1 Department of Immunology and Theranostics, Arthur Riggs Diabetes and Metabolism Research Institute, Beckman Research Institute of the City of Hope, Duarte, CA 91010, USA.; 2 Department of Immuno-Oncology, Beckman Research Institute of the City of Hope, Duarte, CA 91010, USA.; 3 Department of Physiology, David Geffen School of Medicine, University of California, Los Angeles, Los Angeles, CA 90095, USA.; 4 Department of Microbiology, Immunology and Genetics, Ben-Gurion University of Negev, Bear Sheva, Israel.

## Abstract

Steroid nuclear receptor coactivator 2 (SRC2) is a member of a family of transcription coactivators. While SRC1 inhibits the differentiation of regulatory T cells (T_regs_) critical for establishing immune tolerance, we show here that SRC2 stimulates T_reg_ differentiation. SRC2 is dispensable for the development of thymic T_regs_, whereas naive CD4^+^ T cells from mice deficient of SRC2 specific in T_regs_ (*SRC2^fl/fl^/Foxp3^YFP-Cre^*) display defective T_reg_ differentiation. Furthermore, the aged *SRC2^fl/fl^/Foxp3^YFP-Cre^* mice spontaneously develop autoimmune phenotypes including enlarged spleen and lung inflammation infiltrated with IFNγ-producing CD4^+^ T cells. *SRC2^fl/fl^/Foxp3^YFP-Cre^* mice also develop severer experimental autoimmune encephalomyelitis (EAE) due to reduced T_regs_. Mechanically, SRC2 recruited by NFAT1 binds to the promoter and activates the expression of *Nr4a2*, which then stimulates Foxp3 expression to promote T_reg_ differentiation. Members of SRC family coactivators thus play distinct roles in T_reg_ differentiation and are potential drug targets for controlling immune tolerance.

## Teaser

SRC2 controls the scale of immune responses via activating *Nr4a2* gene to promote CD4^+^Foxp3^+^ iT_reg_ differentiation.

## INTRODUCTION

Regulatory T cells (T_regs_) are essential to protect against autoimmune responses, maintain homeostasis, and damp immune responses after clearance of infection (*1*). However, T_regs_ are often found in tumor microenvironment to effectively prevent antitumor immunity (*2*). The essential physiological function of T_regs_ for induction and maintenance of peripheral tolerance is demonstrated by the uncontrollable autoimmunity in mice and human that lack functional T_regs_ due to a mutation in forkhead box P3 (Foxp3) gene (*3*–*5*). Foxp3 is a lineage-specific transcription factor that determines the generation, maintenance, and function of T_regs_ (*6*). Natural T_regs_ develop in the thymus mostly with T cell receptors (TCRs) recognizing self-antigens (*7*, *8*), whereas induced T_regs_ (iT_regs_) are generated from activation of naive CD4^+^ T cells in the presence of transforming growth factor–β (TGFβ) (*6*, *9*). Naive CD4^+^ T cells can also differentiate into inflammatory effector T cells including T helper 1 (T_H_1), T_H_2, and T_H_17 (*10*, *11*), which are inhibited by T_regs_. A fine balance between inflammatory T cells and T_regs_ is required for a functional immune system. Skewing to inflammatory T cells leads to autoimmunity, whereas development of tumor often associates with the dominance of T_regs_. Thus, understanding the mechanisms that regulate the differentiation of naive T cells into inflammatory T cells and T_regs_ facilitates the development of previously unknown immunotherapies for controlling immune responses.

The steroid receptor coactivator (SRC) family consists of three members, SRC1 (or NCOA1), SRC2 (or NCOA2/TIF2/GRIP1), and SRC3 (or NCOA3/pCIP/ACTR/AIB1). Although SRCs do not directly bind to target DNA, they function as coactivators for steroid nuclear receptors and other transcription factors by interacting with them to stimulate gene transcription (*12*). Hence, SRCs are believed to orchestrate transcription programs critical for multiple cellular processes (*12*). However, the function of SRCs in immune system has long been ignored until recently. Our previous study illustrated that SRC1 can reciprocally regulate the differentiation of inflammatory T_H_17 cells and T_regs_ by promoting T_H_17, whereas it inhibits T_reg_ differentiation (*13*). Similar to SRC1, our other research showed that SRC3 promotes T_H_17 differentiation (*14*), which was also demonstrated by an independent study that further indicated the selective role of SRC3 in the differentiation of pathogenic T_H_17 cells (*15*). Therefore, SRC1 and SRC3 have nonredundant function in stimulating T_H_17 differentiation. With regard to T_regs_, a recent report using germline *SRC3^−/−^* mice and SRC3 inhibitor hinted at a possible function of SRC3 in T_regs_ (*16*). However, SRC3 was found dispensable for T_reg_ differentiation using T cell–specific SRC3 knockout mice (*15*). In contrast to SRC1 and SRC3, the function of SRC2 in T cells remains unknown.

Using T_reg_-specific SRC2 knockout mice (*SRC2^fl/fl^/Foxp3^YFP-Cre^*) and T cell–specific SRC2 knockout mice (*SRC2^fl/fl^/CD4^Cre^*), we demonstrated the essential function of SRC2 in the maintenance of immune balance via regulating the generation of iT_regs_. Naive CD4^+^ T cells from *SRC2^fl/fl^/Foxp3^YFP-Cre^* and *SRC2^fl/fl^/CD4^Cre^* mice were defective in T_reg_ differentiation in vitro and in vivo. Consistently, aged *SRC2^fl/fl^/Foxp3^YFP-Cre^* mice displayed enlarged spleens, weight loss, and damaged lung tissues that were infiltrated with lymphocytes producing inflammatory cytokines. In addition, *SRC2^fl/fl^/Foxp3^YFP-Cre^* mice developed more severe EAE associated with reduced T_regs_ and increased inflammatory CD4^+^ T cells. RNA sequencing (RNA-seq) analysis showed that after polarizing under T_reg_ conditions, *SRC2^fl/fl^/Foxp3^YFP-Cre^* CD4^+^ cells had lower levels of Nr4a2, a transcription factor known to directly regulate Foxp3 expression, and forced expression of *Nr4a2* rescued T_reg_ differentiation in both *SRC2^fl/fl^/Foxp3^YFP-Cre^* and *SRC2^fl/fl^/CD4^Cre^* CD4^+^ cells. Mechanistically, SRC2 interacted with NFAT1, and both were recruited to the promoter region of *Nr4a2*. Furthermore, CRISPR-Cas9–mediated deletion of the DNA promoter region that binds SRC2 and NFAT1 reduced Nr4a2 expression and further impaired T_reg_ differentiation. Therefore, SRC2 recruited by NFAT1 stimulates the expression of Nr4a2, which then promotes T_reg_ differentiation via up-regulation of Foxp3. Together with our previously reported negative role of SRC1 in T_reg_ differentiation, different members of SRC family, SRC1 and SRC2, have opposite functions in T_reg_ differentiation.

## RESULTS

### SRC2 is not required for thymic T_reg_ development but is essential for T_reg_ differentiation from naive CD4^+^ T cells

To determine the function of SRC2 in T_regs_, we generated two strains of mice that deleted *Ncoa2* (encoding SRC2) in T_regs_ (*SRC2^fl/fl^/Foxp3^YFP-Cre^*) or T cells (*SRC2^fl/fl^/CD4^Cre^*), respectively. *Ncoa2* gene deletion in T_regs_ of *SRC2^fl/fl^/Foxp3^YFP-Cre^* mice (fig. S1A) and CD4^+^ cells of *SRC2^fl/fl^/CD4^Cre^* mice (fig. S1B) was confirmed by the lack of SRC2 protein assessed by immunoblot. Furthermore, greatly reduced *Ncoa2* mRNA was observed in *SRC2^fl/fl^/Foxp3^YFP-Cre^* CD4^+^ T cells than in *Foxp3^YFP-Cre^* CD4^+^ T cells as early as 20 hours after polarization (fig. S1C), and in *SRC2^fl/fl^/CD4^Cre^* CD4^+^ T cells than in *SRC2^fl/fl^* CD4^+^ T cells (fig. S1D) polarized under T_reg_ differentiation conditions.

**Fig. 1.**
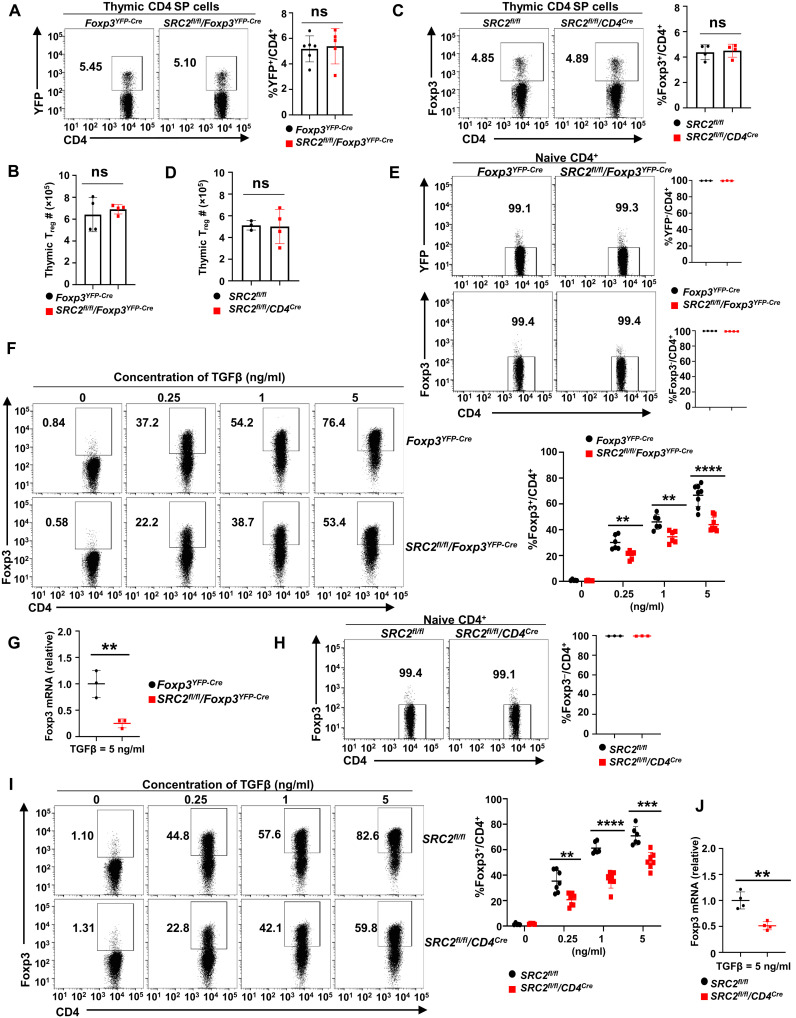
SRC2 is not required for nature T_reg_ development but essential for T_reg_ differentiation from naive Foxp3^−^CD4^+^ T cells. (**A** and **C**) Representative flow cytometric analysis (left panels) and the percentage (right panels) of T_reg_ (YFP^+^ or Foxp3^+^) cells among CD4^+^ thymocytes from indicated mice (*n* ≥ 4 per genotype). (**B** and **D**) Absolute number of T_reg_ in the thymus from indicated mice (*n* ≥ 3 per genotype). (**E**) Representative flow cytometric analysis (left panels) and percentage (right panels) of naive YFP^−^ (top) and Foxp3^−^ (bottom panels) among CD4^+^ cells isolated from spleens of indicated mice (*n* = 3 per genotype). (**F**) Representative flow cytometric analysis (left panels) and percentage (right panel) of Foxp3^+^ T_regs_ differentiated from naive CD4^+^ T cells shown in (E) in the presence of different concentrations of TGFβ for 48 hours (*n* ≥ 4 per treatment cohort). (**G**) Quantitative polymerase chain reaction (qPCR) analysis of *Foxp3* mRNA in indicated CD4^+^ cells 48 hours after T_reg_ differentiation in the presence of TGFβ (5 ng/ml; *n* = 3 per genotype). (**H**) Representative flow cytometric analysis (left panels) and percentage (right panel) of naive Foxp3^−^ CD4^+^ cells isolated from spleens of indicated mice (*n* = 3 per genotype). (**I**) Representative flow cytometric analysis (left panels) and percentage (right panel) of Foxp3^+^ T_regs_ differentiated from naive Foxp3^−^ CD4^+^ cells shown in (H) when treated with varying concentrations of TGFβ for 48 hours (*n* ≥ 5 per genotype). (**J**) qPCR analysis of *Foxp3* mRNA in indicated CD4^+^ cells 48 hours after T_reg_ differentiation in the presence of TGFβ (5 ng/ml; *n* = 4 per genotype). Boxed area: Cell population of interest. Data are from three experiments (B, D, G, J; A, C, E, F, H, and I, right panels; presented as means ± SD) or are from one representative of three independent experiments (A, C, E, F, H, and I, left panels). **P < 0.01; ***P < 0.001; ****P < 0.0005; ns, not significant (two-tailed Student’s *t* test).

Since T_regs_ develop in the thymus, thymic T_reg_ development was first examined. Overall, thymocyte development was normal in *SRC2^fl/fl^/Foxp3^YFP-Cre^* and *SRC2^fl/fl^/CD4^Cre^* mice, as indicated by thymic cellularity (fig. S1, E and G) and percentage of thymocyte subsets: CD4^−^CD8^−^ double-negative (early thymocytes), CD4^+^CD8^+^ double-positive, and CD4^+^/CD8^+^ single-positive (mature T cells) cells compared to *Foxp3^YFP-Cre^* mice (fig. S1F) and *SRC2^fl/fl^* mice (fig. S1H), respectively. There was no significant difference in the percentage and the number of thymic T_regs_ between *Foxp3^YFP-Cre^* and *SRC2^fl/fl^/Foxp3^YFP-Cre^* mice (Fig. 1, A and B) and between *SRC2^fl/fl^* and *SRC2^fl/fl^/CD4^Cre^* mice (Fig. 1, C and D). Thus, SRC2 is not essential for thymocyte development, including thymic natural T_reg_ development.

iT_regs_ are differentiated from peripheral naive CD4^+^ T cells in the presence of TGFβ. Next, we examined the function of SRC2 on iT_reg_ differentiation. *Foxp3^YFP-Cre^* and *SRC2^fl/fl^/Foxp3^YFP-Cre^* mice allow us to use yellow fluorescent protein (YFP) as a reporter for Foxp3 expression (fig. S1I). We confirmed that purified naive CD4^+^YFP^−^ T cells (Fig. 1E, top panels) from spleens of *Foxp3^YFP-Cre^* and *SRC2^fl/fl^/Foxp3^YFP-Cre^* mice were Foxp3^−^ (Fig. 1E, bottom panels). Furthermore, in the presence of TGFβ, these naive CD4^+^YFP^−^ T cells differentiated into Foxp3^+^YFP^+^ T_regs_ (Fig. 1F and fig. S1J). However, the ability of naive CD4^+^YFP^−^ T cells from *SRC2^fl/fl^/Foxp3^YFP-Cre^* mice to generate iT_regs_ was greatly impaired compared to the CD4^+^ T cells from *Foxp3^YFP-Cre^* mice at all the TGFβ concentrations that we tested (Fig. 1F). Consistently, *Foxp3* mRNA was decreased in *SRC2^fl/fl^/Foxp3^YFP-Cre^* CD4^+^ cells than in *Foxp3^YFP-Cre^* CD4^+^ cells after T_reg_ differentiation (Fig. 1G). The observed impaired T_reg_ differentiation was not due to changes in cell proliferation and survival, which were comparable between *Foxp3^YFP-Cre^* and *SRC2^fl/fl^/Foxp3^YFP-Cr^* cells gated on either Foxp3^+^ (fig. S1, K and L, top panels) or Foxp3^−^ cells (fig. S1, K and L, bottom panels), as monitored by the proliferation marker Ki-67 (fig. S1K) and live/dead dye (fig. S1L) at 20 and 48 hours after initiation of T_reg_ differentiation. Similarly, compared to control SRC2*^fl/fl^* CD4^+^ T cells, the capacity of naive *SRC2^fl/fl^/CD4^Cre^* Foxp3^−^CD4^+^ T cells (Fig. 1H) to generate iT_regs_ in vitro was greatly decreased under varying TGFβ concentrations (Fig. 1I) and showed correspondingly lower *Foxp3* mRNA levels in *SRC2^fl/fl^/CD4^Cre^* CD4^+^ cells after T_reg_ differentiation (Fig. 1J). Thus, we show that SRC2, although dispensable for thymic T_reg_ development, is essential for iT_reg_ generation from naive CD4^+^ T cells in vitro.

### SRC2 is required for generating iT_regs_ in vivo

**Fig. 2.**
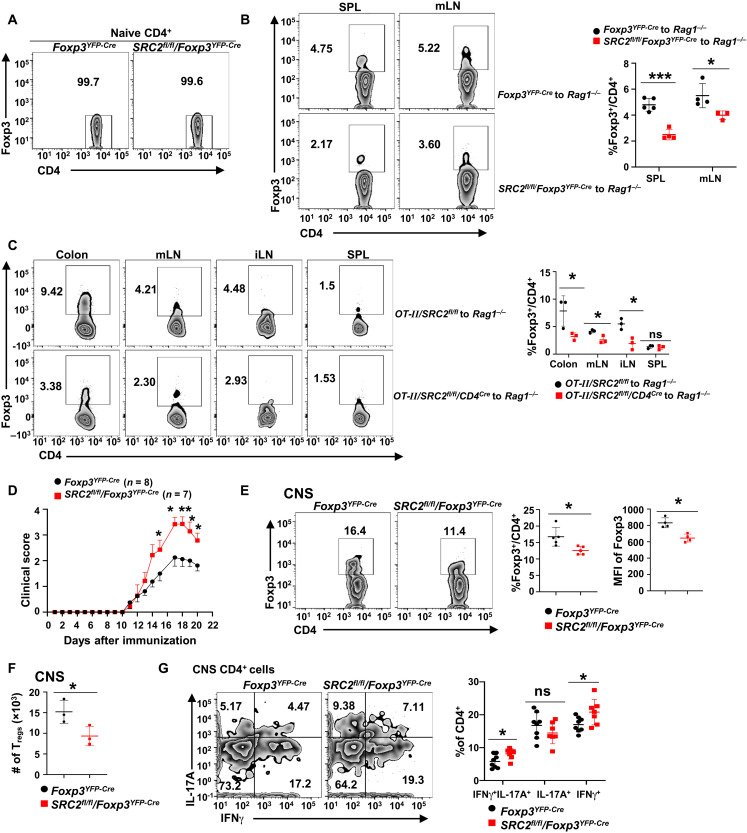
SRC2 is required for generating T_regs_ in vivo. (**A**) Representative flow cytometric analysis of Foxp3^−^ cells in sorted naive CD4^+^ cells from indicated mice before adoptive transfer into *Rag1^−/−^* mice. (**B**) Representative flow cytometric analysis (left panels) and percentage (right panel) of Foxp3^+^CD4^+^ T_regs_ in spleen and mLN of *Rag1^−/−^* mice 3 weeks after adoptive transfer of 0.4 × 10^6^ naive CD4^+^ cells (*n* ≥ 3 per genotype). (**C**) Representative flow cytometric analysis (left panels) and percentage (right panel) of Foxp3^+^ CD4^+^ T_regs_ in colon, mLN, iLN, and spleen of *Rag1^−/−^* mice transferred with 3 × 10^6^ naive *OT-II/SRC2^fl/fl^* or *OT-II/SRC2^fl/fl^/CD4^Cre^* CD4^+^ cells and subsequently treated with OVA (20 mg/ml) for 5 days (*n* = 3 per genotype). (**D**) Mean clinical EAE scores of indicated mice at different days after EAE induction with MOG_35–55_. (**E**) Representative flow cytometric analysis (left panels) and the percentage and Foxp3 MFI (right panels) of Foxp3^+^CD4^+^ T_regs_ recovered from the CNS of EAE-induced mice (*n* = 5 per genotype). (**F**) Number of Foxp3^+^CD4^+^ T_regs_ recovered from the CNS of EAE-induced mice (*n* = 3 per genotype). (**G**) Representative flow cytometric analysis (left panels) and the percentage (right panel) of interferon-γ–positive (IFNγ^+^) and IL-17A^+^ cells among CD4^+^ T cells recovered from the CNS of EAE-induced mice (*n* ≥ 7 per genotype). Boxed area: Cell population of interest. Data are from three experiments (D, presented as means ± SEM; B, C, E, and G, right panels, presented as means ± SD) or are from one representative of three independent experiments (A; B, C, E, and G, left panels). *P < 0.05, **P < 0.01, and ***P < 0.001 (two-tailed Student’s *t* test).

To determine the function of SRC2 in vivo in T_reg_ generation, sorted naive *Foxp3^YFP-Cre^* or naive *SRC2^fl/fl^/Foxp3^YFP-Cre^* YFP^−^CD4^+^ T cells that lack Foxp3^+^ T_regs_ (Fig. 2A) were adoptively transferred to *Rag1^−/−^* mice (*17*, *18*). Significant T_regs_ were detected in spleens and mesenteric lymph nodes (mLNs) 3 weeks after adoptive transfer of naive *Foxp3^YFP-Cre^* CD4^+^ cells (Fig. 2B, top two panels). In contrast, naive *SRC2^fl/fl^/Foxp3^YFP-Cre^* CD4^+^ T cells generated less T_regs_ in vivo (Fig. 2B, bottom). Next, an oral tolerance model was used to determine SRC2 function in the generation of T_regs_ in vivo (*9*). In this model, sorted naive CD4^+^Foxp3^−^ T cells from *OT-II/SRC2^fl/fl^* or *OT-II*/*SRC2^fl/fl^/CD4^Cre^* mice (fig. S2A) were adoptively transferred into *Rag1^−/−^* mice, and T_regs_ were induced mostly in gut-associated lymphoid tissues by feeding ovalbumin peptide (OVA) in drinking water (Fig. 2C). Consistently, significantly less T_regs_ were generated from *OT-II*/*SRC2^fl/fl^/CD4^Cre^* CD4^+^ T cells than from *OT-II/SRC2^fl/fl^* CD4^+^ T cells in colon, mLN, and inguinal lymph nodes (iLNs), but no difference was observed in spleens. Collectively, these results demonstrate an essential role for SRC2 in promoting T_reg_ differentiation in vivo.

To determine whether SRC2-regulated generation of T_reg_ plays a role in controlling autoimmune responses, we compared the development of EAE between *Foxp3^YFP-Cre^* and *SRC2^fl/fl^/Foxp3^YFP-Cre^* mice (Fig. 2D). Compared to *Foxp3^YFP-Cre^* mice, *SRC2^fl/fl^/Foxp3^YFP-Cre^* mice developed much severer EAE and had significantly less T_regs_ with lower levels of Foxp3 (Fig. 2, E and F), but more inflammatory CD4^+^IFNγ^+^IL-17A^+^ and CD4^+^IFNγ^+^ cells but not interleukin-17A–positive (IL-17A^+^) cells in the central nervous system (CNS) (Fig. 2G and fig. S2B for gating strategy). Our results thus support an essential function for SRC2 in T_reg_ differentiation in vivo, and SRC2-regulated generation of T_regs_ controls the scale of immune responses in vivo.

### Aged *SRC2^fl/fl^/Foxp3^YFP-Cre^* mice develop inflammation-associated lung tissue damages

**Fig. 3.**
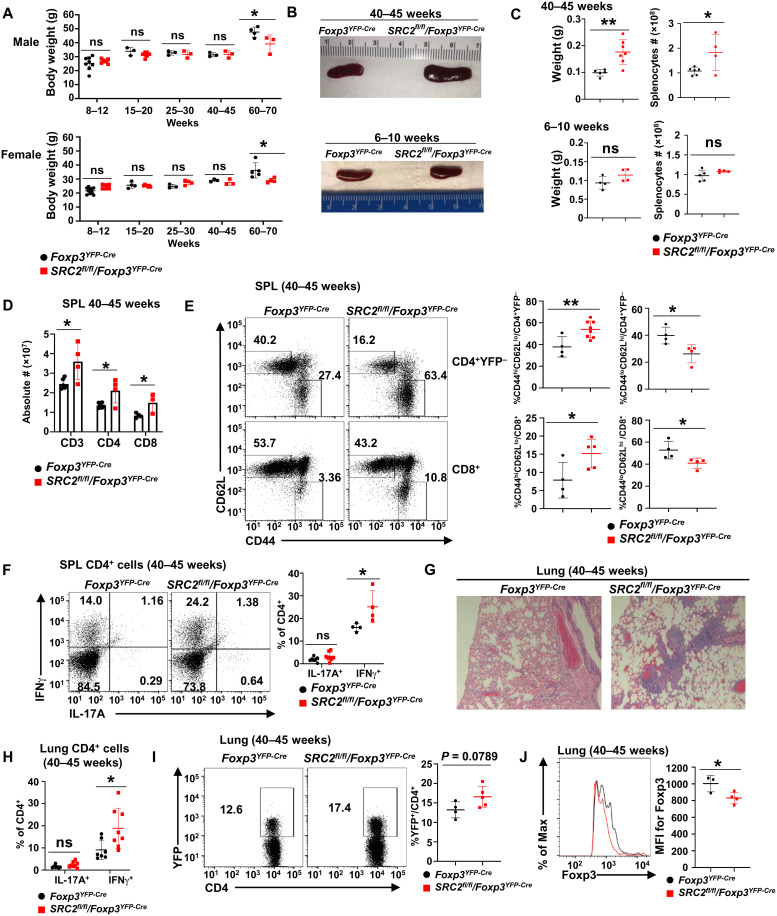
Aged *SRC2^fl/fl^/Foxp3^YFP-Cre^* mice develop inflammation-associated lung tissue damages. (**A**) Body weight measurement of indicated male (top panel) and female (bottom panel) mice at different ages (*n* ≥ 3 per genotype per group). (**B**) Representative image of spleens from older (top panel) and younger (bottom panel) mice of indicated genotypes. (**C**) Weight (left panels) and cellularity (right panels) of the spleens from older (top panels) and younger (bottom panels) indicated mice (*n* ≥ 4 per genotype per group). (**D**) Absolute number of CD3^+^, CD4^+^, and CD8^+^ T cells in the spleens from indicated older mice (*n* ≥ 4 per genotype per group). (**E**) Representative flow cytometric analysis (left panels) and percentage (right panels) of CD62L and CD44 cells among splenic CD4^+^YFP^−^ or CD8^+^ T cells from indicated aged mice (*n* ≥ 4 per genotype per group). (**F**) Representative flow cytometric analysis (left panels) and percentage (right panel) of IL-17A^+^ and IFNγ^+^ cells among CD4^+^ cells from spleens of indicated aged mice (*n* ≥ 4 per genotype). (**G**) Section of hematoxylin and eosin (H&E)–stained lung from indicated aged mice. (**H**) Percentage of IL-17A^+^ and IFNγ^+^ cells among CD4^+^ cells recovered from lung of indicated aged mice (*n* ≥ 7 per genotype). (**I**) Representative flow cytometric analysis (left panels) and percentage (right panel) of T_regs_ (YFP^+^) among CD4^+^ cells recovered from lung of indicated aged mice (*n* ≥ 4 per genotype). (**J**) Representative flow cytometric analysis (left panel) and the MFI (right panel) for Foxp3 in Foxp3^+^CD4^+^ cells recovered from lung of indicated aged mice (*n* ≥ 3 per genotype). Boxed area: Cell population of interest. Data are from three experiments (A, C, D, and H; E, F, I, and J, right panels; presented as means ± SD) or are from one representative of three independent experiments (B and G; E, F, I, and J, left panels). *P < 0.05 and **P < 0.01 (two-tailed Student’s *t* test).

We noticed that aged *SRC2^fl/fl^/Foxp3^YFP-Cre^* mice were smaller and suffered from hair loss compared to their age-matched *SRC2^fl/fl^* counterparts (fig. S3A). Sixty- to 70-week-old *SRC2^fl/fl^/Foxp3^YFP-Cre^* mice suffered modest weight loss than *Foxp3^YFP-Cre^* mice, regardless of sex, compared to younger mice (Fig. 3A). In addition, 40- to 45-week-old *SRC2^fl/fl^/Foxp3^YFP-Cre^* mice had enlarged spleen (splenomegaly) (Fig. 3B), which was further confirmed by increased spleen weight and cellularity (Fig. 3C) when compared to age-matched *Foxp3^YFP-Cre^* mice (Fig. 3, B and C), whereas no differences in spleen size, weight, and cellularity were observed between the cohorts at 6- to 10-week-old mice (Fig. 3, B and C). Increased numbers of CD3^+^ T cells, including both CD4^+^ and CD8^+^ T cell subsets, contributed to the increased cellularity of the spleens from older *SRC2^fl/fl^/Foxp3^YFP-Cre^* mice (Fig. 3D). Since splenomegaly is a sign of inflammation, it indicates that the inflammatory disease resulted from defective T_regs_ because of specific deletion of SRC2 in aged *SRC2^fl/fl^/Foxp3^YFP-Cre^* mice. The percentage of CD44^hi^CD62^lo^ memory-like cells were significantly increased, while CD44^lo^CD62^hi^ naive CD4^+^ and CD8^+^ T cells were significantly decreased in spleens of 40- to 45-week-old *SRC2^fl/fl^/Foxp3^YFP-Cre^* mice (Fig. 3E) than in *Foxp3^YFP-Cre^* mice; no differences in these cell subtypes were observed in spleens at 6 to 10 weeks between the cohorts (fig. S3B). At 40 to 45 weeks, *SRC2^fl/fl^/Foxp3^YFP-Cre^* mice had increased percentage of IFNγ^+^CD4^+^ cells but not IL-17A^+^CD4^+^ splenocytes (Fig. 3F). Furthermore, compared to *Foxp3^YFP-Cre^* mice, *SRC2^fl/fl^/Foxp3^YFP-Cre^* mice had severe lung damage (Fig. 3G) associated with increased inflammatory IFNγ^+^CD4^+^ cells (Fig. 3H) at 40 to 45 weeks. Increased T_regs_ (Fig. 3I) were found in the damaged lung of *SRC2^fl/fl^/Foxp3^YFP-Cre^* mice. However, these *SRC2^fl/fl^/Foxp3^YFP-Cre^* T_regs_ had significantly reduced mean florescence intensity (MFI) for Foxp3 than *Foxp3^YFP-Cre^* T_regs_ (Fig. 3J). Increased T_regs_ were also found in spleens and lymph nodes of older (40 to 45 weeks) *SRC2^fl/fl^/Foxp3^YFP-Cre^* mice with obvious lung inflammation but not in younger mice (6 to 25 weeks) (fig. S3C), suggesting that increased T_regs_ in older *SRC2^fl/fl^/Foxp3^YFP-Cre^* mice are likely a compensatory mechanism trying to inhibit the observed inflammation. This is consistent with what is observed in other mice with defective T_regs_ (*19*). Consistent with reduced MFI for Foxp3 found in T_regs_ from lungs of older *SRC2^fl/fl^/Foxp3^YFP-Cre^* mice, MFI for Foxp3 in T_regs_ in the spleens and lymph nodes showed the same trend of decrease with increase in age of *SRC2^fl/fl^/Foxp3^YFP-Cre^* mice compared to *Foxp3^YFP-Cre^* mice (fig. S3D). Analysis of CD62L^hi^ cells, a marker for naive T_regs_, indicated a significant decrease in CD62L^hi^ naive T_regs_ in the spleen, lymph nodes, and lungs of older *SRC2^fl/fl^/Foxp3^YFP-Cre^* mice (fig. S3, E and F), supporting the notion that increased T_regs_ in older *SRC2^fl/fl^/Foxp3^YFP-Cre^* mice likely result from T_reg_ proliferation (*20*). These results demonstrate the critical function of SRC2 in T_reg_-dependent maintenance of immune tolerance in vivo.

### SRC2 is dispensable for the suppressive function of T_regs_ in younger mice

**Fig. 4.**
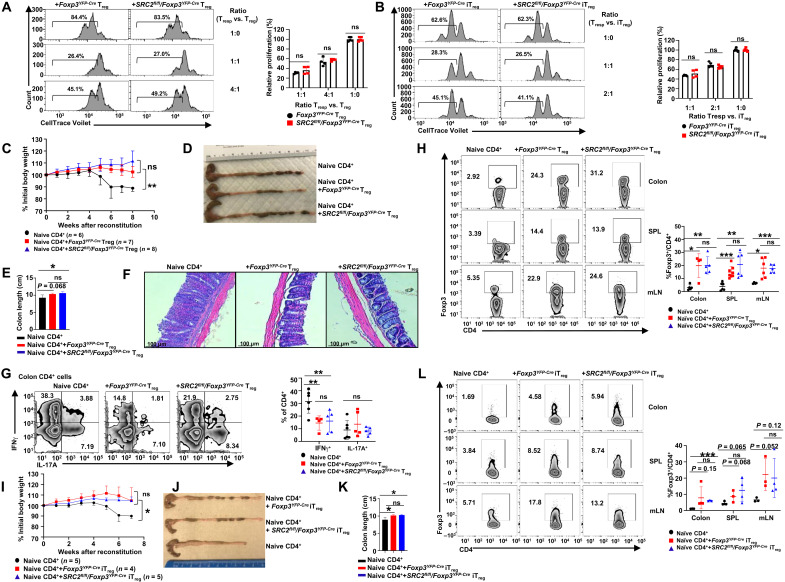
SRC2 is dispensable for the suppressive function of T_regs_ in younger mice. (**A** and **B**) Representative flow cytometric analysis (left panels) and the relative proliferation (right panel) of responder T (T_resp_) cells cultured with splenic YFP^+^CD4^+^ T_regs_ isolated from indicated 6- to 8-week-old mice (A) or YFP^+^CD4^+^ iT_regs_ differentiated in vitro (B) (*n* = 4 per genotype). (**C**) Body weight of *Rag1^−/−^* recipients over time after adoptive transfer of WT CD45RB^hi^CD25^−^CD4^+^ naive T cells alone or in combination with splenic YFP^+^CD4^+^ T_regs_ from 6- to 8-week-old mice. (**D** to **F**) Representative image of colons (D), colon length (E) (*n* = 5 per genotype), and H&E-stained colon section (F) from *Rag1^−/−^* recipients 8 weeks after adoptive transfer. (**G**) Representative flow cytometric analysis (left panels) and percentage (right panel) of CD4^+^IL-17A^+^ and CD4^+^IFNγ^+^ cells recovered from colons of *Rag1^−/−^* recipients (*n* ≥ 4 per group). (**H**) Representative flow cytometric analysis (left panels) and percentage (right panel) of Foxp3^+^CD4^+^ T_regs_ recovered from colon, spleen, and mLN of *Rag1^−/−^* recipients (*n* ≥ 4 per group). (**I**) Body weight of *Rag1^−/−^* recipients over time after adoptive transfer of WT CD45RB^hi^CD25^−^CD4^+^ naive T cells alone or in combination with in vitro differentiated YFP^+^CD4^+^ iT_regs_. (**J** and **K**) Representative image of colons (J) and colon length (K) (*n* ≥ 4 per genotype) from *Rag1^−/−^* recipients 7 weeks after adoptive transfer. (**L**) Representative flow cytometric analysis (left panels) and percentage (right panel) of Foxp3^+^CD4^+^ T_regs_ recovered from colon, spleen, and mLN of *Rag1^−/−^* recipients (*n* ≥ 4 per group). Boxed area: Cell population of interest. Data are from three experiments (C and I, presented as means ± SEM; E, I, K, A, B, G, H, and L, right panels, presented as means ± SD) or are from one representative of three independent experiments (D, F, and J; A, B, G, H, and L, left panels). *P < 0.05, **P < 0.01, and ***P < 0.001 (two-tailed Student’s *t* test).

We first examined the expression of several surface markers, CD73, CD39, CD25, and CTLA-4, which are indicators for the suppressive function of T_regs_ (*21*–*23*). There were no significant differences in the expression for all these markers between *Foxp3^YFP-Cre^* and *SRC2^fl/fl^/Foxp3^YFP-Cre^* T_regs_ (CD4^+^YFP^+^) from spleens and mLNs in 6- to 8-week-old mice (fig. S4A). To determine the suppressive function of T_regs_, we assessed the ability of T_regs_ to inhibit CD4^+^ T cell proliferation in vitro. SRC2 deficiency did not alter the suppressive function of CD4^+^YFP^+^ T_regs_ sorted from the spleen of 6- to 8-week-old *SRC2^fl/fl^/Foxp3^YFP-Cre^* mice or in vitro derived *SRC2^fl/fl^/Foxp3^YFP-Cre^* iT_regs_ (Fig. 4, A and B), suggesting that SRC2 is not essential for the suppressive function of T_regs_. Last, the in vivo function of T_regs_ was tested in the prevention of colitis. In the absence of T_regs_, adoptive transfer of naive CD4^+^ T cells (CD45RB^hi^CD25^−^CD4^+^) into *Rag1^−/−^* mice induced severe colitis, as indicated by weight loss (Fig. 4C), shortened colon (Fig. 4, D and E), damaged intestinal tissues (Fig. 4F), and greatly increased proinflammatory IFNγ^+^CD4^+^ T cells in the colon (Fig. 4G) and mLN (fig. S4B). In contrast, cotransfer of CD4^+^YFP^+^ *Foxp3^YFP-Cre^* T_regs_ or *SRC2^fl/fl^/Foxp3^YFP-Cre^* T_regs_ with naive CD4^+^ T cells rescued these severe colitis phenotypes in *Rag1^−/−^* mice (Fig. 4, C to F). In these rescued mice, we also observed a significant reduction in proinflammatory IFNγ^+^CD4^+^ T cells in the colon and mLN (Fig. 4G and fig. S4B). Furthermore, higher levels of adoptively transferred T_regs_ from either *Foxp3^YFP-Cre^* or *SRC2^fl/fl^/Foxp3^YFP-Cre^* mice were found in the gut-associated tissues and spleens of recipients (Fig. 4H), thus contributing to the prevention of colitis.

Similar to natural T_regs_ sorted from younger mice, in vitro derived iT_regs_ also prevented colitis; *Foxp3^YFP-Cre^* and *SRC2^fl/fl^/Foxp3^YFP-Cre^* iT_regs_ showed comparable inhibitory efficacy in preventing weight loss (Fig. 4I) and shortening of the colon (Fig. 4, J and K). Adoptively transferred iT_regs_ derived from either *Foxp3^YFP-Cre^* or *SRC2^fl/fl^/Foxp3^YFP-Cre^* naive CD4^+^ T cells were maintained at comparable levels in the colon, spleen, and mLN of the recipients (Fig. 4L). These results suggest that SRC2, although required for T_reg_ differentiation, is not essential for suppressive T_reg_ function.

Since aged *SRC2^fl/fl^/Foxp3^YFP-Cre^* mice showed inflammation, an indication of defective T_reg_ function, we thus determined the inhibitory function of T_regs_ from older mice. T_regs_ from 26-week-old *SRC2^fl/fl^/Foxp3^YFP-Cre^* mice started to show slightly decreased inhibitory activity at 4:1 T_resp_ (responder T cell)/T_reg_ ratio than those from age-matched *Foxp3^YFP-Cre^* mice (fig. S4C), suggesting that the impaired inhibitory function of T_regs_ from older *SRC2^fl/fl^/Foxp3^YFP-Cre^* mice likely also contributes to the observed lung tissue inflammation.

### SRC2 stimulates the expression of *Nr4a2* critical for T_reg_ differentiation

**Fig. 5.**
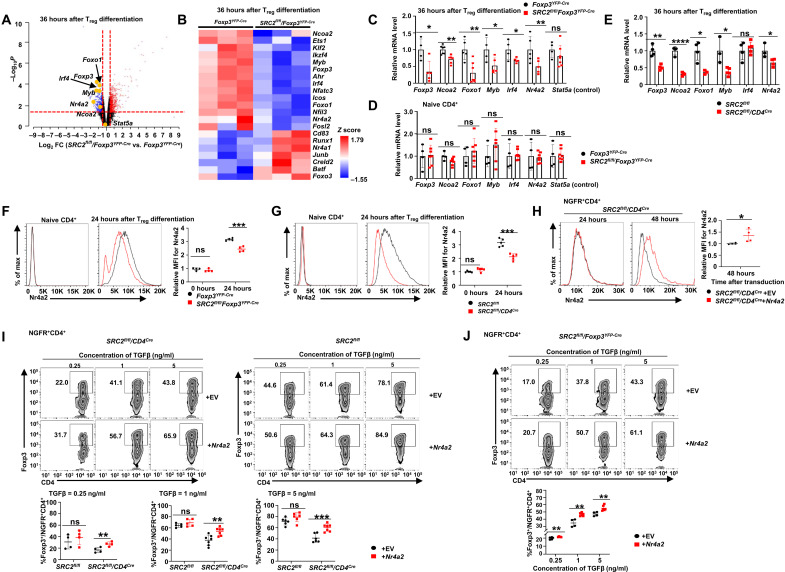
SRC2 stimulates the expression of Nr4a2 critical for T_reg_ differentiation. (**A**) Volcano plot comparison of gene expression between *Foxp3^YFP-Cre^* and *SRC2^fl/fl^/Foxp3^YFP-Cre^* CD4^+^ cells (*n* = 3 per genotype). Differentially up-regulated genes (red) and down-regulated genes (blue) with a cutoff at *P* < 0.05 and fold change (FC) > 1.4 are shown. (**B**) Heatmap of *Ncoa2* and other genes known to regulate T_reg_ differentiation. (**C** to **E**) qPCR analysis of *Foxp3, Ncoa2, Foxo1, Myb, Irf4, Nr4a2,* and *Stat5a* (control) mRNA in CD4^+^ cells 36 hours after T_reg_ polarization (C and E) and naive CD4^+^ cells (D) derived from indicated mice (*n* ≥ 4 per genotype per group). (**F** and **G**) Representative flow cytometric analysis of protein levels (left panels) and relative MFI (right panel) for Nr4a2 in indicated genotypes of naive CD4^+^ cells versus CD4^+^ cells 24 hours after T_reg_ polarization [*n* = 4 per genotype for (F) and *n* = 5 per genotype for (G)]. (**H**) Representative flow cytometric analysis of protein levels (left panels) and relative MFI (right panel) for Nr4a2 in *SRC2^fl/fl^/CD4^Cre^* CD4^+^ T cells transduced with retrovirus expressing ± *Nr4a2* and differentiated under T_reg_ polarization conditions for 24 or 48 hours (*n* = 4 per genotype). (**I** and **J**) Representative flow cytometric analysis (top panels) and the percentage (bottom panels) of Foxp3^+^CD4^+^ T_regs_ among indicated genotypes of NGFR^+^CD4^+^ cells transduced with retrovirus expressing ± *Nr4a2* and polarized for 48 hours under T_reg_ conditions (*n* ≥ 4 per genotype per group). EV, empty vector; NGFR, marker of transduction; boxed region, cell population of interest. Data are from three experiments (A to H, right panels; I and J, bottom panels; presented as means ± SD) or are from one representative of three independent experiments (F to H, left panels; I and J, top panels). *P < 0.05, **P < 0.01, ***P < 0.001, and ****P < 0.0005 (two-tailed Student’s *t* test).

We next determined mechanisms for SRC2-regulated T_reg_ differentiation. We first excluded the function of SRC2 in the regulation of Foxp3 stability, as the degradation rate of Foxp3 in *Foxp3^YFP-Cre^* and *SRC2^fl/fl^/Foxp3^YFP-Cre^* T_regs_ was equivalent (fig. S5A). SRC2 is a transcriptional coactivator that is believed to regulate cellular function by controlling gene expression. Thus, we next performed RNA-seq analysis to detect the transcriptome of the following four groups of cells (fig. S5B, left panel): (i) *Foxp3^YFP-Cre^* naive CD4^+^ T cells, (ii) *SRC2^fl/fl^/Foxp3^YFP-Cre^* naive CD4^+^ T cells, (iii) *Foxp3^YFP-Cre^* CD4^+^ cells polarized in TGFβ for 36 hours, and (iv) *SRC2^fl/fl^/Foxp3^YFP-Cre^* CD4^+^ cells polarized in TGFβ for 36 hours. The expression of Foxp3 was significantly lower in polarized *SRC2^fl/fl^/Foxp3^YFP-Cre^* CD4^+^ than the wild-type (WT) control (fig. S5B, middle panel). Principal components analysis of transcriptomes clustered three repeats within each group together, whereas the naive CD4^+^ T cells and polarized CD4^+^ cells showed the biggest differences in gene expression patterns (fig. S5B, right panel), indicating the excellent quality and reproducibility of RNA-seq results. Comparing transcriptomes between differentiated *Foxp3^YFP-Cre^* and *SRC2^fl/fl^/Foxp3^YFP-Cre^* cells, we identified many differentially expressed genes known to regulate T_reg_ differentiation (Fig. 5, A and B, and fig. S5C). Not surprisingly, *Ncoa2* (encoding SRC2) was among the most down-regulated genes due to gene deletion. *Foxp3* was also down-regulated in *SRC2^fl/fl^/Foxp3^YFP-Cre^* cells, confirming impaired T_reg_ differentiation.

Since SRC2 is a transcriptional coactivator, we paid particular attention to the down-regulated transcription factor genes. Four transcription factors—*Myb, Irf4, Foxo1*, and *Nr4a2—*were down-regulated in the absence of SRC2 and are known to positively regulate T_reg_ differentiation (*9*, *24*–*26*). Individual quantitative polymerase chain reaction (qPCR) confirmed the down-regulation of these four transcription factors together with *Ncoa2* and *Foxp3* in *SRC2^fl/fl^/Foxp3^YFP-Cre^* CD4^+^ T_regs_, whereas *Stat5a* served as a control that did not show any significant changes in qPCR analysis (Fig. 5C). These four transcription factors did not show obvious differential mRNA expression between *Foxp3^YFP-Cre^* and *SRC2^fl/fl^/Foxp3^YFP-Cre^* in naive CD4^+^ T cells (Fig. 5D), suggesting that the observed changes were induced upon T_reg_ differentiation. *Ncoa2* also did not show differences between *Foxp3^YFP-Cre^* and *SRC2^fl/fl^/Foxp3^YFP-Cre^* naive CD4^+^ T cells, as its gene deletion is only induced when Foxp3 starts to express during T_reg_ differentiation. Our results suggest that SRC2 is required for the up-regulation of these four transcription factors in T_regs_ at the mRNA level. Since *SRC2^fl/fl^/CD4^Cre^* CD4^+^ T cells also showed impaired T_reg_ differentiation, we compared the expression of above transcription factors between differentiated *SRC2^fl/fl^* and *SRC2^fl/fl^/CD4^Cre^* CD4^+^ cells (Fig. 5E). Except *Irf4*, the other three transcription factors were also down-regulated together with *Foxp3* in *SRC2^fl/fl^/CD4^Cre^* CD4^+^ cells after polarization under T_reg_ conditions. Therefore, SRC2 stimulates the expression of some transcription factors critical for T_reg_ differentiation.

We next evaluated the effects of forced expression of above down-regulated transcription factors on T_reg_ differentiation from CD4^+^ T cells that are deficient in SRC2. For this purpose, retrovirus expressing individual transcription factor was transduced into *SRC2^fl/fl^/CD4^Cre^* CD4^+^ T cells that then differentiated into T_regs_ in the presence of TGFβ. Forced expression of *Foxo1, Irf4*, or *Myb* together with green fluorescent protein (GFP) did not rescue T_reg_ differentiation in *SRC2^fl/fl^/CD4^Cre^* CD4^+^ T cells compared to the cells transduced with virus expressing only GFP [empty vector (EV); fig. S5D]. Examining transcription factor protein expression, Foxo1 (fig. S5E) and interferon regulatory factor 4 (IRF4; fig. S5F) protein levels were not obviously down-regulated upon T_reg_ differentiation in the absence of SRC2 (Myb protein could not be reliably detected).

Consistent with prior reports that *Nr4a2* is up-regulated upon T cell activation (*27*, *28*), polarized *Foxp3^YFP-Cre^* (Fig. 5F, middle panel) and *SRC2^fl/fl^* (Fig. 5G, middle panel) CD4^+^ cells in the presence of TGFβ expressed higher levels of Nr4a2 at 24 hours than their corresponding respective naive CD4^+^ counterparts without stimulation (Fig. 5, F and G, left panel). However, after 24-hour polarization under T_reg_ conditions, Nr4a2 in both *SRC2^fl/fl^/Foxp3^YFP-Cre^* (Fig. 5F, middle panel) and *SRC2^fl/fl^/CD4^Cre^* (Fig. 5G, middle panel) CD4^+^ cells failed to up-regulate to the levels detected in their corresponding WT counterparts *Foxp3^YFP-Cre^* or *SRC2^fl/fl^* CD4^+^ cells, respectively. This result suggests that SRC2 can promote *Nr4a2* expression. Retrovirus expressing *Nr4a2* together with nerve growth factor receptor (NGFR) was used to transduce *SRC2^fl/fl^* CD4^+^ cells (Fig. 5H); this greatly increased Nr4a2 levels at 48 hours, but not 24 hours, after transduction when compared to control cells transduced with EV expressing only NGFR (Fig. 5H). Correspondingly, transduction with Nr4a2 significantly elevated Foxp3 expression in CD4^+^ cells at 48 hours, but not 24 hours, after transduction and differentiation compared to EV control cells (fig. S5G). Moreover, forced expression of Nr4a2 significantly stimulated T_reg_ differentiation in *SRC2^fl/fl^/CD4^Cre^* (Fig. 5I, left panels), but not in *SRC2^fl/fl^* (Fig. 5I, right panels), CD4^+^ T cells at all concentrations of TGFβ that we tested, suggesting that exogenous Nr4a2 is able to overcome SRC2 deficiency and rescue the T_reg_ differentiation defect. In addition, forced expression of Nr4a2 was also able to stimulate the differentiation of T_regs_ from *SRC2^fl/fl^/Foxp3^YFP-Cre^* CD4^+^ cells (Fig. 5J). However, Nr4a2, together with IRF4 (fig. S5H) or Foxo1 (fig. S5I), did not further stimulate T_reg_ differentiation compared to Nr4a2 alone in *SRC2^fl/fl^/CD4^Cre^* CD4^+^ T cells. Our results thus support a model that SRC2 promotes T_reg_ differentiation via up-regulation of Nr4a2, which is known to stimulate *Foxp3* gene expression (*26*).

### SRC2 recruited by NFAT1 binds to the promoter and activates gene expression of *Nr4a2*

**Fig. 6.**
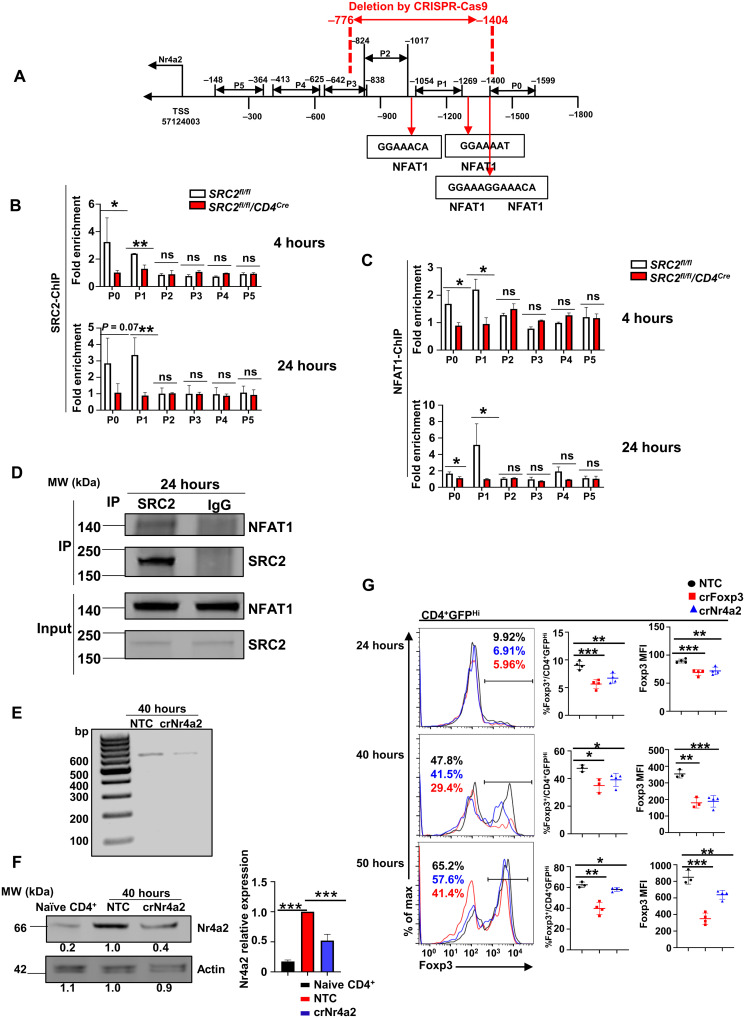
SRC2 recruited by NFAT1 binds to the promoter and active gene expression of *Nr4a2*. (**A**) Schematic representation of the six regions on *Nr4a2* promoter covered by P0 to P5 primers, the locations of identified NFAT1-binding sites, and the region deleted using CRISPR-Cas9. (**B** and **C**) ChIP-qPCR analysis of SRC2 binding (B) or NFAT1 binding (C) to the *Nr4a2* promoter (P0 to P5 regions) in CD4^+^ cells from indicated genotypes under T_reg_ polarization at the indicated time points (*n* ≥ 3 per genotype per group). (**D**) Immunoblot analysis of NFAT1 among anti-SRC2 antibody immunoprecipitated (IP) SRC2 complexes from *SRC2^fl/fl^* CD4^+^ cells 24 hours after T_reg_ polarization. Bottom blots, whole-cell lysate input control. (**E**) PCR analysis of the abundance of NFAT1/SRC2 binding region on *Nr4a2* promoter in CD4^+^ cells transduced with virus expressing nontarget control (*NTC*) or the region containing NFAT1/SRC2-binding guiding RNAs shown in (A) (*crNr4a2*) and polarized under T_reg_ condition for 40 hours. (**F**) Immunoblot analysis of Nr4a2 in naive CD4^+^ cells or CD4^+^ T cells transduced with virus expressing *NTC* and *crNr4a2* shown in (E) and polarized under T_reg_ conditions for 40 hours. The number in the bottom of the blots is the relative mean intensity of each band, and the right panel is the summary of the relative mean intensity. (**G**) Representative flow cytometric analysis of Foxp3 (left panels), percentages of Foxp3^+^ cells (middle panels), and MFI for Foxp3 (right panels) among GFP^hi^CD4^+^ cells transduced with virus expressing *NTC, crFoxp3*, and *crNr4a2* guiding RNAs and polarized under T_reg_ condition at the indicated time points (*n* ≥ 3 per treatment per group). Data are from three experiments (B, C, and F, right panels; G, middle and right panels; presented as means ± SD) or are from one representative of three independent experiments (D to G, left panels). *P < 0.05, **P < 0.01, and ***P < 0.001 (two-tailed Student’s *t* test). MW, molecular weight; bp, base pairs.

We next determined how SRC2 regulates *Nr4a2* expression. Since *Nr4a2* mRNA is decreased in the absence of SRC2, we hypothesized that SRC2 is critical for the transcriptional up-regulation of *Nr4a2* expression. SRC2 ChIP-seq (chromatin immunoprecipitation and DNA sequencing) analysis detected stronger DNA binding signals at the *Nr4a2* promoter region in *SRC2^fl/fl^* CD4^+^ cells than in *SRC2^fl/fl^/CD4^Cre^* CD4^+^ cells after 36-hour polarization under T_reg_ conditions (fig. S6A). Several pairs of primers were then designed to cover the 1.6-kb promoter region (P0 to P5) upstream of *Nr4a2* gene transcription starting site (Fig. 6A). ChIP assays with these primers detected signals indicating interaction of SRC2 at the distal promoter elements (P0 and P1) but not at the proximal P3 to P5 regions at 4 hours (Fig. 6B, top panel) and increasingly at 24 hours (Fig. 6B, bottom panel) after T_reg_ differentiation. As a coactivator, SRC2 does not directly bind but is recruited to DNA by transcription factors. We thus searched for potential transcription factor–binding sites in the promoter region by PROMO, a virtual laboratory for the identification of putative transcription factor binding sites (TFBS) in DNA sequences from a species or groups of species of interest, and identified a few transcription factor–binding sites including signal transducer and activator of transcription 4 (Stat4), NFAT, and Stat6 (fig. S6B). Several conserved NFAT1-binding sites surrounding the P1 region (Fig. 6A) drew our attention, as NFAT1 is a known regulator of T_reg_ differentiation (*29*, *30*) and has been reported to stimulate *Nr4a2* expression in CD8^+^ cells (*31*). Thus, ChIP assays were performed using the same P0 to P5 primers (Fig. 6A and fig. S6A) to determine whether NFAT1 interacts with *Nr4a2* promoter. NFAT1-binding signals were detected at the distal P0 and P1 regions at 4 hours (Fig. 6C, top panel) and increasingly at P1 at 24 hours (Fig. 6C, bottom panel) after T_reg_ differentiation. This was similar to the SRC2-binding patterns, suggesting that NFAT1 recruits SRC2 to the *Nr4a2* promoter. Furthermore, NFAT1 was also detected in anti-SRC2 antibody immunoprecipitated complexes from *SRC2^fl/fl^* CD4^+^ cells 24 hours after T_reg_ differentiation (Fig. 6D and fig. S6C for full blot image).

To further determine whether the NFAT1/SRC2 binding is functionally important for T_reg_ differentiation via *Nr4a2* regulation, the region containing the potential NFAT1/SRC2-binding sites was deleted using CRISPR-Cas9 with two guiding RNAs (crNr4a2) in CD4^+^ T cells from mice expressing Cas9 (Fig. 6A and fig. S6D). At the same time, we used a nontargeting construct (NTC) as a negative control and a *Foxp3* gene deletion construct (crFoxp3) as a positive control. Deletion of the region containing NFAT1/SRC2-binding sites was confirmed in crNr4a2-transduced cells by PCR analysis (Fig. 6E). Nr4a2 levels were greatly higher in differentiated cells (NTC control) than in naive CD4^+^ T cells (Fig. 6F and fig. S6E for full blot image), confirming our previous observation that Nr4a2 is up-regulated during T_reg_ differentiation (Fig. 5, F and G). Furthermore, Nr4a2 levels were reduced significantly following deletion of the region containing NFAT1/SRC2-binding sites compared to the NTC control (Fig. 6F and fig. S6E for full blot image), supporting the notion that NFAT1/SRC2 recruitment to the *Nr4a2* promoter stimulates gene expression. Compared to NTC control, Foxp3 expression was down-regulated using the crFoxp3 gene deletion construct at 24, 40, and 50 hours after transduction and T_reg_ differentiation (Fig. 6G), confirming successful deletion of *Foxp3* gene with CRISPR-Cas9. Deletion of the NFAT1/SRC2-binding region on *Nr4a2* promoter by crNr4a2 led to impaired T_reg_ differentiation in terms of both percentage of Foxp3^+^ T_regs_ (Fig. 6G, middle panels) and MFI of Foxp3 (Fig. 6G, right panels), as compared to that of NTC control. As an additional control, we also deleted an adjacent DNA fragment (fig. S6, F and G), which did not affect *Nr4a2* expression (fig. S6H) and also did not affect Foxp3 differentiation (fig. S6I). Therefore, *Nr4a2* promoter region that binds SRC2/NFAT1 is a critical regulatory element that controls *Nr4a2* expression and T_reg_ differentiation. Together, NFAT1 recruits SRC2 to *Nr4a2* locus to stimulate *Nr4a2* expression, which then activates *Foxp3* expression, resulting in the promotion of T_reg_ differentiation.

## DISCUSSION

SRC2 has long been known to have an anti-inflammatory function in the innate immune system (*32*). Nuclear factor κB (NF-κB) and activating protein 1 (AP1) are the transcription factors responsible for the activation of the majority of the inflammatory genes in response to the stimulus by inflammatory signals and cytokines (*33*). By acting as a cofactor for glucocorticoid receptor (GR) that is tethered to the promoter regions by protein-protein interaction with NF-κB or AP1, SRC2 is able to inhibit the expression of such inflammatory genes (*34*, *35*). This partially explains the immune inhibitory effects of GR that is the target of broadly used immunosuppressive drugs in clinics. In addition, GR can also constrain inflammation by sequestrating SRC2 from IRFs that use SRC2 as coactivator (*36*). IRFs are the critical transcription factors stimulating the expression of the inflammatory genes including chemokines and cytokines in response to type I interferons (IFNs) (*37*). When SRC2 is sequestrated by GR, IRFs, in the absence of its coactivator, fail to stimulate their target genes, resulting in the suppression of inflammation (*38*, *39*). Furthermore, SRC2 also inhibits the expression of the genes encoding inflammatory cytokines, tumor necrosis factor–α (TNFα), IL-6, and IL-1, when working with estrogen receptors (*40*–*42*). Our novel findings suggest that SRC2 suppresses immune responses by promoting the generation of iT_regs_, which is an important regulatory component of adaptive immunity. T_regs_ repress the function of effector T cells by direct contact via inhibitory surface molecules PD-1 and CTLA-4 and by production of anti-inflammatory cytokines including TGFβ and IL-10 (*43*). These anti-inflammatory cytokines also impair innate immune responses. Therefore, SRC2 functions in both innate and adaptive immune cells to balance the overall immune responses by promoting the inhibitory arm of the immunity.

Three members of SRC family coactivators are all approximately 160 kDa in size, share overall similar structure, and are very conservative in amino acid sequence (*44*, *45*). They often act as coactivators for the same transcription factors. However, we previously showed that SRC1 inhibited T_reg_ differentiation. In contrast, we show here that SRC2 stimulates iT_reg_ differentiation, indicating that different members of SRC family could have totally opposite functions in T_regs_. Upon phosphorylation by TCR signaling molecule protein kinase C–θ (PKC-θ), SRC1 dissociates Foxp3 from Foxp3-RORγt complexes, resulting in accelerated degradation of Foxp3 (*13*). Thus, a posttranslational phosphorylation event of SRC1 is the critical mechanism for SRC1-inhibited T_reg_ differentiation. Here, we show that by acting as a coactivator for NFAT1, SRC2/NFAT1-mediated transcription activation of *Nr4a2* stimulates the Foxp3 gene expression, resulting in promoting T_reg_ differentiation. Therefore, SRC2-controlled transcription program is critical for T_reg_ differentiation. Together, our results demonstrated that the function of SRCs is not solely dependent on their associated transcription factors; the highly conserved members of SRC family can control diverse function via regulating gene expression in a context-dependent manner: (i) SRCs recruit different cofactors including epigenetic modification enzymes, allowing formation of distinct transcriptional complexes. (ii) SRCs are subjected to different posttranslational modifications including phosphorylation, ubiquitination, sumoylation, acetylation, and methylation, which regulates SRC-interacting proteins and/or stability (*46*). (iii) SRCs sense different environmental cues that instruct SRCs to make corresponding changes. Our results thus demonstrate how different members of SRCs, although highly conserved, can control the same function such as T_reg_ differentiation via distinct mechanisms, resulting in totally different outcomes.

We showed that *Nr4a2* (*Nurr1*) is a critical target gene of SRC2 in the regulation of T_reg_ differentiation. Consistent with this result, both SRC2 and Nr4a2 are selectively required for T_reg_ differentiation from naive T cells but are not required for natural T_reg_ development in the thymus (*27*, *28*). A previous study showed that Nr4a2 is essential for the inhibitory function of T_regs_ (*27*). However, the inhibitory function of both natural T_regs_ from younger mice and iT_regs_ was not impaired when SRC2 was deleted in T_regs_ in our current study. This is likely due to lower but not complete absence of Nr4a2 expression in *SRC2^fl/fl^/Foxp3^Cre-YFP^* T cells, and these relatively lower levels of Nr4a2 are sufficient for the inhibitory function but not for the differentiation of iT_regs_. Furthermore, T_regs_ from older, but not younger, *SRC2^fl/fl^/Foxp3^Cre-YFP^* mice start to display impaired inhibitory function, suggesting that the SRC2 function in T_regs_ is age dependent and the impaired inhibitory function of T_regs_ likely contributes to the observed lung tissue inflammation in old *SRC2^fl/fl^/Foxp3^Cre-YFP^* mice. This impaired inhibitory function of T_regs_ may result from the accumulation of the defects with growing age, which is worthy of further investigation. Nr4a2 is an orphan nuclear receptor that lacks a classical ligand-binding pocket and thus functions as a ligand-independent transcription factor (*28*). Nr4a2 activity is therefore believed to be largely regulated by its expression. We found that Nr4a2 is up-regulated by SRC2, which likely results in increased Nr4a2 activity required for T_reg_ differentiation. We showed that SRC2 physically interacts with NFAT1, and both bind to the same promoter region of *Nr4a2* gene during T_reg_ differentiation. Ca^2+^/calcineurin/NFAT signals activated by TCR stimulation are reported to be required for up-regulating Nr4a2 gene expression in T cells (*31*, *47*). Thus, our results support a model that SRC2 associated with NFAT1 is recruited to the Nr4a2 promoter to stimulate the expression of Nr4a2, which, in turn, promotes T_reg_ differentiation by activating *Foxp3* gene expression. SRC2 can be recruited by multiple transcription factors; thus, it is worth investigating whether SRC2 can also be recruited by other transcription factors to coordinate the overall transcription program essential for T_reg_ differentiation.

Increased iT_regs_ in tumor microenvironment are responsible for failed antitumor immune responses (*48*). Checkpoint inhibitors that disrupt the function of T_regs_ via blocking inhibitory PD-1 and CTLA-4 have shown efficacy in the treatment of cancers. Intensive research has been focused on preventing the function and/or reducing the number of T_regs_ for boosting immune responses against tumors. Our results show that SRC2 stimulates the generation of iT_regs_, and thus, inhibition of SRC2, similar to SRC2 knockout mice, is expected to reduce the number of T_regs_. Therefore, SRC2 is a potential target for boosting antitumor immunity. All members of SRC family are considered oncogenes, as they play roles in tumorigenesis in different types of cancers (*49*, *50*). Small-molecule SRC inhibitors have already been developed for the treatment of cancers (*51*, *52*). It would be interesting to test whether SRC2 inhibitors can boost antitumor immunity by preventing the generation of T_regs_. Together with our results, it raises the possibility that SRC2 inhibitors can be used to treat cancers by targeting both cancer cells and immune system.

## MATERIALS AND METHODS

### Mice

Transgenic *CD4^Cre^* (*TgCd4^cre^,* 022071), *Rag1^−/−^* (*Rag1^tm1Mom^*, 002216), *Cas9* (*Rosa26^LSL-Cas9^*, 028551), and *C57BL* (*B6*, 000664) mice were purchased from the Jackson Laboratory. *SRC2^fl/fl^* mice were obtained from Jianming Xu Lab (Molecular and Cell Biology, Baylor College of Medicine, TX). *OT-II* mice were obtained from Jianhua Yu Lab (Department of Hematology and Hematopoietic Cell Transplantation, City of Hope, CA), and *Foxp3^YFP-Cre^* mice were obtained from Mark Boldin Lab (Molecular and Cellular Biology, Beckman Research Institute, City of Hope, CA). All mice were bred at the C57BL/6j background and housed under specific pathogen–free conditions in the Animal Resource Center at the Beckman Research Institute of City of Hope under protocols approved by the Institutional Animal Care and Use Committee (IACUC#07023). Mice were 10 to 12 weeks of age for EAE studies and 6 to 10 weeks of age for other experiments, unless indicated otherwise, with littermates age- and sex-matched across experimental groups.

### Antibodies and cytokines

Monoclonal antibodies against mouse CD3 (145-2C11), CD28 (37.51), IL-4 (11B11), and IFNγ (XMG1.2), as well as phycoerythrin (PE)–conjugated anti-CD8 (dilution ratio, 1:100; 53-6.7), allophycocyanin (APC)–conjugated anti-Foxp3 (dilution ratio, 1:100; FJK-16s), PE-indotricarbocyanine (Cy7)–conjugated anti–IL-17A (dilution ratio, 1:100; eBio17B7), APC-conjugated anti-IFNγ (dilution ratio, 1:100; XMG1.2), and LIVE/DEAD Fixable Near-IR Dead Cell Stain (dilution ratio, 1:1000; L34976) were from Invitrogen. PE-conjugated anti-CD25 (dilution ratio, 1:100; PC61), Brilliant Violet (BV) 605–conjugated anti-CD4 (dilution ratio, 1:100; RM4-5), BV 421–conjugated anti-CD3 (dilution ratio, 1:100; 145-2C11), APC-conjugated anti-CD45 (dilution ratio, 1:100; I3/2.3), PE-Cy7–conjugated anti-CD45RB (dilution ratio, 1:100; C363-16A), PE-conjugated anti–Ki-67 (dilution ratio, 1:100; 16A8), PE-Cy7–conjugated anti-CD62L (dilution ratio, 1:100; MEL-14), APC-Cy7–conjugated anti-CD44 (dilution ratio, 1:100; IM7), Alexa Fluor 488–conjugated anti-IRF4 (dilution ratio, 1:100; IRF4.3E4), PE-Cy7–conjugated anti-NGFR (dilution ratio, 1:100; ME20.4), PE-conjugated anti-CD73 (dilution ratio, 1:100; TY/11.8), PE-Cy7–conjugated anti-CD39 (dilution ratio, 1:100; Duha59), APC-Cy7–conjugated anti-CD45 (dilution ratio, 1:100; 30-F11), PE-Cy7–conjugated anti–CTLA-4 (dilution ratio, 1:100; UC10-4B9), and recombinant murine IL-2 were from BioLegend. APC-conjugated anti-CD25 (dilution ratio, 1:100; PC61) was from BD. Fluorescein isothiocyanate (FITC)–conjugated anti-Nr4a2 (dilution ratio, 1:150; orb464231) was from Biorbyt. FITC-conjugated anti-Foxo1 (dilution ratio, 1:100; 83N7F8) was from Novus. PE-conjugated anti–IL-10 (dilution ratio, 1:100; JES5-16E3) was from eBioscience. Rabbit anti-hamster antibody (55398) was from MP Biomedicals. Antibodies against SRC2 (dilution ratio, 1:2000; A300-346A, Bethyl), rabbit IgG (P120-101, Bethyl), NFAT1 (dilution ratio, 1:1000; 5861S, Cell Signaling Technology), Nr4a2 (dilution ratio, 1:150; sc-376984, Santa Cruz Biotechnology), and β-actin (dilution ratio, 1:1000; SC-8422, Santa Cruz Biotechnology) were used for immunoblot analysis. Recombinant mouse TGFβ was from Miltenyi Biotec.

### Plasmids

The retroviral vector murine stem cell virus (MSCV)–internal ribosomal entry site (IRES)–GFP was a gift from W. S. Pear (University of Pennsylvania). Complementary DNA (cDNA) encoding *Foxo1* was cloned into MSCV-IRES-GFP vector. IRF4-MIEG-GFP was a gift from Mark H. Kaplan Lab (Indiana University School of Medicine), and MSCV-HA-Nr4a2-IRES-NGFR was a gift from Joyce Chen Lab (La Jolla Institute for Immunology, La Jolla, CA, USA). MSCV-IRES-NGFR (plasmid #27489), MSCV-PIG-Myb (plasmid #66988), and retro–guide RNA (gRNA)–eGFP (plasmid #116926) were purchased from Addgene.

### Flow cytometry

For surface staining, cells isolated from mice or in vitro culture were directly stained with antibodies and/or fixable live/dead dye with 2% fetal bovine serum (FBS) and 1 mM EDTA at 4°C for 15 min. For transcription factor staining, cells prestained with surface markers were fixed and permeabilized in TF Fix/Perm buffer (BD Biosciences) at 4°C for 20 min, washed once with TF Perm/Wash buffer, and stained with target markers in the TF Perm/Wash buffer at 4°C for 15 min. For intracellular cytokine analysis, cells were stimulated with phorbol 12-myristate 13-acetate (50 ng/ml; Sigma-Aldrich) and ionomycin (750 ng/ml; Sigma-Aldrich) at 37°C for 3 hours in the presence of GolgiStop (BD Biosciences) before staining. After stimulation, cells were stained with surface markers and then fixed and permeabilized with Cytofix/Cytoperm buffer (BD Biosciences) for 20 min followed by staining cytokines in the Perm/Wash buffer (BD Biosciences) after washing. The expression of surface and intracellular markers was analyzed with a BD LSRFortessa flow cytometer.

### Isolation of naive CD4^+^ T cells and in vitro T_reg_ differentiation

Naive CD4^+^ T cells were isolated from mouse spleens by negative selection using the Naive CD4^+^ T Cell Isolation Kit (Miltenyi Biotec). Suspensions of 5 × 10^5^ cells per milliliter of RPMI 1640 medium (Corning Inc.) containing 2 mM l-glutamine, 50 μM β-mercaptoethanol, penicillin (100 U/ml), streptomycin (100 mg/ml), and 10% FBS (Corning Inc.) were cultured in 24-well plates or 48-well plates precoated with rabbit anti-hamster (0.1 mg/ml). The medium was supplemented with hamster anti-CD3 (0.25 μg/ml), hamster anti-CD28 (1 μg/ml), TGFβ (0.25, 1, or 5 ng/ml), anti–IL-4 (2.5 μg/ml), and anti-IFNγ (2.5 μg/ml) for T_reg_ differentiation for up to 48 hours.

### In vivo induction of iT_regs_ by adoptively transferring naive CD4^+^ cells

Splenic cells were collected from *Foxp3^YFP-Cre^* or *SRC2^fl/fl^/Foxp3^YFP-Cre^* mice (6 to 8 weeks). Naive CD4^+^ T cells were first enriched by negative selection using the Naive CD4^+^ T Cell Isolation Kit, and then CD4^+^YFP^−^ cells were sorted via FACSAria Fusion (BD) to enable a high purity of ≥99.0%. A total of 4 × 10^5^ naive CD4^+^YFP^−^ cells were intraperitoneally injected into sex-matched *Rag1^−/−^* mice. Three weeks after adoptive transfer, cells from spleen and mLN of *Rag1^−/−^* recipient mice were collected and analyzed.

### In vivo induction of iT_regs_ by oral tolerance

Splenic cells were collected from *OT-II*/*SRC2^fl/fl^* or *OT-II*/*SRC2^fl/fl^/CD4^Cre^* mice (6 to 8 weeks), naive CD4^+^ T cells were first enriched by negative selection using the Naive CD4^+^ T Cell Isolation Kit, and CD4^+^CD25^−^ cells were then sorted via FACSAria Fusion to enable a high purity of ≥99.0%. A total of 3 × 10^6^ cells were intraperitoneally injected to sex-matched *Rag1^−/−^* mice. After 24 hours, recipient mice were provided with grade VI OVA (20 mg/ml; Sigma-Aldrich) ad libitum in drinking water for 5 days. Drinking water containing OVA was changed every 2 days. Cells were collected from colon, spleen, iLN, and mLN at day 6 for analysis.

### Induction and assessment of EAE

EAE was induced and assessed according to the manufacturer’s instructions (Hooke Laboratories, Lawrence, MA). Briefly, *Foxp3^YFP-Cre^* or *SRC2^fl/fl^/Foxp3^YFP-Cre^* mice were immunized with 200 mg of MOG_35–55_ (Hooke Laboratories) in complete Freund’s adjuvant by subcutaneous injection at two dorsal sites of mice, followed by two intraperitoneal injections of 80 ng of pertussis toxin at days 0 and 1. The severity of EAE was monitored and evaluated on a scale from 0 to 5 according to Hooke Laboratories’ guideline. Briefly, 0 represents no disease. 1 represents paralyzed tail. 2 represents hindlimb weakness. 3 represents hindlimb paralysis. 4 represents hindlimb and forelimb paralysis, and 5 represents moribund and death. When a mouse was euthanized because of severe paralysis, a score of 5 was entered for that mouse for the rest of the experiment.

### In vivo T_reg_ suppression assay

Colitis was induced in sex-matched *Rag1^−/−^* mice by intraperitoneally injecting 4 × 10^5^ CD45RB^hi^CD25^−^CD4^+^ naive T cells sorted from the spleen of C57BL mice (8 to 10 weeks). For natural T_reg_ suppression assay, 2 × 10^5^ CD4^+^YFP^+^ T_regs_ sorted from the spleen of 6- to 8-week-old *Foxp3^YFP-Cre^* or *SRC2^fl/fl^/Foxp3^YFP-Cre^* mice were mixed with 4 × 10^5^ CD45RB^hi^CD25^−^CD4^+^ naive T cells from C57BL mice and injected into sex-matched *Rag1^−/−^* mice. For iT_reg_ suppression assay, iT_regs_ were first induced in vitro from naive CD4^+^ cells from *Foxp3^YFP-Cre^* or *SRC2^fl/fl^/Foxp3^YFP-Cre^* mice for 48 hours, and CD4^+^YFP^+^ iT_regs_ were sorted out. In total, 2 × 10^5^ CD4^+^YFP^+^ iT_regs_ were mixed with 4 × 10^5^ CD45RB^hi^CD25^−^CD4^+^ naive T cells from C57BL mice and injected to sex-matched *Rag1^−/−^* mice as above. Mice were weighed immediately following T cell transfer and weekly thereafter. Seven to 8 weeks after cell transfer, colon, spleen, and mLN were removed from *Rag1^−/−^* recipient mice for analysis.

### In vitro T_reg_ suppression assay

Sorted CD4^+^CD25^−^ T cells were labeled with CellTrace Violet (C34557, Invitrogen) and served as T_resp_ cells. T_resp_ cells (6 × 10^5^ cells/ml) were cocultured with CD4^+^YFP^+^ T_regs_ sorted from the spleens of *Foxp3^YFP-Cre^* or *SRC2^fl/fl^/Foxp3^YFP-Cre^* mice in 48-well plates [precoated with rabbit anti-hamster (0.1 mg/ml)] in culture medium supplemented with hamster anti-CD3 (0.25 μg/ml), hamster anti-CD28 (1 μg/ml), and IL-2 (20 ng/ml) for 3 days. For iT_reg_, naive CD4^+^ cells were purified and cultured under the T_reg_ differentiation condition for 48 hours, and CD4^+^YFP^+^ iT_regs_ were sorted and cocultured with T_resp_ cells for 3 days. The ratios of T_resp_ cells to T_regs_ were 1:0, 1:1, and 4:1 for T_regs_ sorted from mice and 1:0, 1:1, and 2:1 for iT_regs_ sorted from in vitro differentiation. Proliferation of T_resp_ cells was assessed by flow cytometry.

### Histology study

Tissues were cleaned and fixed with 4% paraformaldehyde, embedded in paraffin, and then sectioned and stained with hematoxylin and eosin.

### RNA-seq and analysis

Naive CD4^+^ T cells isolated from *Foxp3^YFP-Cre^* or *SRC2^fl/fl^/Foxp3^YFP-Cre^* mice were differentiated into T_regs_ in 24-well plates in the presence of TGFβ (5 ng/ml), anti–IL-4, and anti-IFNγ for 36 hours. Naive CD4^+^ T cells and CD4^+^ cells after 36 hours of T_reg_ differentiation were collected and subjected to RNA extraction with the RNeasy Mini Kit (QIAGEN). Each group has three replicates from different mice. Quality control, library preparation, and sequencing were performed at Novogene. The analysis was performed through Partek Flow. Briefly, the sequence reads were aligned to the mouse whole genome (GRCm38) with validation of quality through prealignment and postalignment quality assurance (QA)/quality control (QC). Aligned reads were further subjected to quantification using the Partek E/M algorithm and normalization to counts per million (CPM) with 0.001 added to each. The identification of differentially expressed features was performed through the Partek GSA algorithm that applies multiple statistical models to each gene. Genes with total counts over 10 were considered to be statistically expressed in the cells.

### Reverse transcription quantitative real-time PCR

Total RNA of cells was extracted according to the manufacturer’s guide using the RNeasy Mini Kit (QIAGEN). The first-strand cDNA synthesis was performed by reverse transcription using a Tetro cDNA synthesis kit (Bioline). Subsequent qPCR was performed using PowerUp SYBR Green Master Mix (Applied Biosystems) in the QuantStudio 3 Real-Time PCR System (Thermo Fisher Scientific). The primers used for qPCR are listed in table S1. The amplification efficiency of all primers has been tested, and the optimized conditions were used in all qPCRs. Gene expression was calculated with the ∆∆*C*_t_ method normalized to the control gene encoding β-actin, and all measurements were performed in triplicate.

### Retroviral transduction

Vectors were firstly transfected to Platinum-E (Plat-E; Cell Biolabs) retroviral packaging cells by using BioT transfection reagent (Bioland Scientific) followed by a changing fresh medium at 24 hours. The virus-containing medium collected at 48 and 72 hours was filtered with a 0.45-μm polyvinylidene difluoride (PVDF) syringe filter (Millipore), followed by either direct transduction to T cells or storing at −80°C for later use. Naive CD4^+^ cells were activated by hamster anti-CD3 (0.25 μg/ml) and hamster anti-CD28 antibodies (1 μg/ml) in precoated plates for 20 hours before transduction. Transduction to activated CD4^+^ T cells was performed by spin infection with viral supernatants (2500*g*, 30°C for 2 hours) in the presence of polybrene (10 μg/ml; Sigma-Aldrich). Afterward, the plates were kept in the incubator at 37°C for 3 hours. The viral supernatant was replaced by a fresh culture medium with polarizing cytokines and antibodies for T_reg_ differentiation.

### Chromatin immunoprecipitation and DNA sequencing

ChIP was performed with the ChIP-IT High Sensitivity Kit (Active Motif, 53040). Briefly, a total of 2 × 10^7^ CD4^+^ cells from *SRC2^fl/fl^* or *SRC2^fl/fl^/CD4^Cre^* after T_reg_ differentiation were fixed and sheared as described in the ChIP-IT High Sensitivity manual. ChIP reactions were then performed on 30 μg of the prepared chromatin using specific antibodies (anti-SRC2 from Bethyl or anti-NFAT1 from Cell Signaling Technology) overnight, followed by precipitation with protein G agarose beads. DNA was recovered for sequencing or quantitative reverse transcription PCR to quantify specific DNA fragments that were precipitated. For sequencing, ChIP-enriched samples were sequenced on NovaSeq PE100 at TGen. The analysis was performed through Partek Flow. Briefly, the sequence reads were aligned to the mm10 mouse genome with validation of quality through prealignment and postalignment QA/QC. The enrichment of SRC2 binding sites across the genome was analyzed using MACS2. The primers used for RT-qPCR are listed in table S1.

### Western blotting and immunoprecipitation

For Western blotting, cells were lysed in radioimmunoprecipitation assay buffer containing 20 mM tris-HCl (pH 7.4), 150 mM NaCl, 1 mM Na_2_EDTA, 1 mM EGTA, 1% NP-40, 1% sodium deoxycholate, 2.5 mM sodium pyrophosphate, 1 mM β-glycerophosphate, 1 mM Na_3_VO_4_, and leupeptin (1 μg/ml) on ice for 45 min and spun down at 15,000 rpm for 10 min at 4°C to collect the extract. The 2× Laemmli sample buffer (Bio-Rad) containing β-mercaptoethanol was mixed with cell extract and heated at 95°C for 5 min. Protein was separated by SDS–polyacrylamide gel electrophoresis (SDS-PAGE) and transferred to PVDF membrane (Millipore). Target proteins were sequentially immunoblotted with relevant primary antibodies and fluorescent secondary antibodies (LI-COR Biosciences) followed by measuring fluorescent intensity with LI-COR Odyssey blot imager (LI-COR Biosciences).

For immunoprecipitation, 1 × 10^7^ cells were lysed in 300 μl of Pierce IP Lysis Buffer (Invitrogen) containing 1% Triton X-100, 20 mM tris-Cl (pH 7.4), 150 mM NaCl, and 5 mM EDTA supplemented with protease inhibitor cocktail (Sigma-Aldrich) on ice for 45 min and spin down at 15,000 rpm for 10 min at 4°C to collect the extract. Five percent of the cell lysate was saved for pre-IP samples. Cell lysates were incubated overnight with the relevant antibodies, and proteins were immunoprecipitated for an additional 4 hours at 4°C with protein A/G Sepharose beads (Millipore). Beads were washed twice by phosphate-buffered saline and by lysis buffer for the last wash. Beads were then suspended in 2× Laemmli sample buffer containing β-mercaptoethanol and heated at 95°C for 5 min. The supernatant containing precipitated proteins was subjected to SDS-PAGE and analyzed by immunoblot.

### Deletion of SRC2/NFAT1-binding region on *Nr4a2* promoter by CRISPR-Cas9

The CRISPR-Cas9 system was used for deleting the SRC2/NFAT1-binding region on mouse *Nr4a2* promoter. Pairs of primers containing sequences of nontargeting control single guide RNAs (sgRNAs), sgRNAs targeting mouse Foxp3, and sgRNAs targeting the upstream or downstream of SRC2/NFAT1-binding region on *Nr4a2* promoter were designed and cloned into retro-gRNA-eGFP vector. Plasmids containing sgRNAs targeting the upstream or downstream of SRC2/NFAT1-binding region on *Nr4a2* promoter were used together to generate retrovirus (crNr4a2) to delete the target region in CD4^+^ cells. Similarly, retroviruses were also produced with the plasmids containing nontargeting control sgRNAs (NTC) or sgRNAs targeting mouse Foxp3 (crFoxp3) to serve as a negative and a positive control to monitor the knocking down of Foxp3 upon infection. To confirm the deletion of SRC2/NFAT1-binding region on *Nr4a2* promoter, the genomic DNA from infected CD4^+^ cells was extracted and the SRC2/NFAT1-binding region abundance was assessed by PCR. As a control, we also deleted an adjacent DNA fragment (crNeg) on *Nr4a2* promoter using the same approach. The sgRNA sequences for NTC, crNr4a2, crFoxp3, and crNeg and the primers used to evaluate the abundance of targeted deletion fragment on *Nr4a2* promoter from genomic DNA are listed in table S1.

### Statistics and reproducibility

The results were analyzed for statistical significance with unpaired Student’s *t* test or one-way analysis of variance (ANOVA) where appropriate. All data are presented as means ± SD. *P* values are calculated using GraphPad Prism and presented where the statistical significance (*P* < 0.05) was found.

## Notes

View/request a protocol for this paper from *Bio-protocol*.
