# Supplementary material for: Steroid nuclear receptor coactivator 2 controls immune tolerance by promoting induced Treg differentiation via up-regulating Nr4a2
Source: Sci Adv. 2022 Jun 15;8(24):eabn7662. doi: 10.1126/sciadv.abn7662 (PMC9200286; doi:10.1126/sciadv.abn7662)
Supplement: Supplementary file 1 — Figs. S1 to S6 Table S1 [file sciadv.abn7662_sm.pdf]

Science Advances
AAAS

Supplementary Materials for

Steroid nuclear receptor coactivator 2 controls immune tolerance by promoting induced T_{reg} differentiation via up-regulating Nr4a2

Wencan Zhang *et al.*

Corresponding author: Zuoming Sun, zsun@coh.org

Sci. Adv. **8**, eabn7662 (2022)
DOI: 10.1126/sciadv.abn7662

This PDF file includes:

Figs. S1 to S6
Tables S1

Fig. S1. SRC2 is not required for thymic Treg development but essential for Treg differentiation from naïve CD4+ T cells.

A Immunoblot analysis of SRC2 in $CD4^+YFP^+$ Tregs isolated from the spleen of *Foxp3*$^{YFP\text{-}Cre}$ and *SRC2*$^{fl/fl}$/*Foxp3*$^{YFP\text{-}Cre}$ mice. **B** Immunoblot analysis of SRC2 in $CD4^+$ cells isolated from the spleen of *SRC2*$^{fl/fl}$ and *SRC2*$^{fl/fl}$/*CD4*^{Cre} mice. **C** qPCR analysis of *Ncoa2* mRNA in indicated $CD4^+$ T cells differentiated under Treg polarization conditions for different times (n≥3 per genotype). **D** qPCR analysis of *Ncoa2* mRNA in indicated $CD4^+$ T cells differentiated under Treg polarization conditions for 48 hours (n=4 per genotype). **E, G** Thymic cellularity quantified using Cellometer (n=3-4 per genotype). **F, H** *Left two panels*: representative flow cytometric analysis of CD4 and CD8 on the surface of thymocytes. *Right panel*: summary of the percentage of $CD4^-CD8^-$ double negative (DN), $CD4^+CD8^+$ double positive (DP), CD4 single positive (SP) and CD8 single positive (SP) thymocytes for individual mice (n≥5 per genotype). Numbers indicate the percentage of the cells in gated area (throughout). **I** Representative flow cytometric analysis of $CD4^+$ T cells from *Foxp3*$^{YFP\text{-}Cre}$ mice for YFP and Foxp3 expression, indicating that $CD4^+YFP^+$ cells are $Foxp3^+$ Tregs whereas $CD4^+YFP^-$ cells are $Foxp3^-$. **J** Representative flow cytometric analysis (left panels) and percentage (right panel) of YFP^+ iTreg induced from indicated naïve $CD4^+$ cells shown in figure 1**I** in the presence of 5ng/ml TGFβ for 48 hours (n≥5 per genotype). **K** Representative flow cytometric analysis (left panels) and percentage (right panel) of $Ki67^+$ cells among indicated genotypes of $CD4^+$ cells 20 hours and 48 hours post Treg differentiation gated on $Foxp3^+$ (top panels) or $Foxp3^-$ (bottom panels) (n≥3 per genotype per group). **L** Representative flow cytometric analysis (left panels) and percentage (right panels) of live cells among indicated $CD4^+$ cells 20 hours and 48 hours post Treg differentiation gated on $Foxp3^+$ (top panels) or $Foxp3^-$ (bottom panels) (n≥3 per genotype per group). Boxed region: cell population of interest. Data are from three experiments (**C**, **D, E, G**; **F, H, J, K, L**, right panels; presented as mean ± s.d.) or are from one representative of three independent experiments (**A**, **B, I**; **F**, **H**, **J, K, L**, left panels). *P<0.05; **P<0.01; **** P<0.0005; ns, not significant (two-tailed Students' *t*-test).

Fig. S2. SRC2 is required for generating induced Tregs *in vivo.*

A Representative flow cytometric analysis (left panels) and percentage (right panel) of sorted naïve $Foxp3^-CD4^+$ cells from *OT-Ⅱ/SRC2*$^{fl/fl}$ or *OT-Ⅱ/SRC2*$^{fl/fl}$/*CD4*^{Cre} mice prior to adoptive transfer to *Rag1*$^{-/-}$ mice (n=4 per genotype). **B** Gating strategy for lymphocytes recovered from central nervous system (CNS) of EAE-induced mice shown in figure 2**D**-2**G**. Boxed region: cell population of interest.

Fig. S3. Aged *SRC2*$^{fl/fl}$/*Foxp3*$^{YFP\text{-}Cre}$ mice develop inflammation-associated lung tissue damages.

A Representative picture of aged *Foxp3*$^{YFP\text{-}Cre}$ and *SRC2*$^{fl/fl}$/*Foxp3*$^{YFP\text{-}Cre}$ mice. **B** Representative flow cytometric analysis (top panels) and percentage (bottom panels) of CD62L and CD44 in splenic $CD4^+YFP^-$ or $CD8^+$ T cells from 6-10 weeks *Foxp3*$^{YFP\text{-}Cre}$ and *SRC2*$^{fl/fl}$/*Foxp3*$^{YFP\text{-}Cre}$ mice (n≥4 per genotype). **C** Percentage of Treg cells (YFP^+) among $CD4^+$ cells recovered from SPL, iLN and mLN of indicated genotypes of mice at different ages (n≥4 per genotype per group). **D** Representative flow cytometric analysis (top panels) and the MFI (bottom panels) for Foxp3 among $CD4^+YFP^+$ cells recovered from SPL, iLN and mLN of indicated genotypes of mice at different ages (n≥4 per genotype per group). **E** Representative flow cytometric analysis (left

panels) and percentage (right panels) of CD62L among YFP$^+$CD4$^+$ Treg cells recovered from SPL, iLN, mLN and lung from indicated genotypes of aged mice (n≥3 per genotype). **F** Absolute number of YFP$^+$CD62L^{hi} cells in SPL from indicated genotypes of aged mice shown in **E**. Boxed region: cell population of interest. Data are from three experiments (**C; B, D,** bottom panels; **E,** left panels; presented as mean ± s.d.), two experiments (**F**; presented as mean ± s.d.) or are from one representative of three independent experiments (**A; B, D,** top panels; **E,** left panels). *P<0.05; **P<0.01; ns, not significant (two-tailed Students' t-test).

Fig. S4. SRC2 is dispensable for the suppressive function of Tregs in younger mice.

A Representative flow cytometric analysis (left panels) and the MFI (right panels) of Treg suppressive functional markers (CD73, CD39, CD25 and CTLA-4) among CD4$^+$YFP$^+$ cells from the spleens (top panels) and mLN (bottom panels) of 6-8 weeks *Foxp3*$^{YFP\text{-}Cre}$ and *SRC2*$^{fl/fl}$/*Foxp3*$^{YFP\text{-}Cre}$ mice (n≥4 per genotype). **B** Representative flow cytometric analysis (left panels) and percentage (right panel) of IL-17A$^+$ and IFNγ$^+$ among CD4$^+$ cells recovered from mLN of *Rag1*$^{-/-}$ recipients eight weeks after receiving CD45RB^{hi}CD25$^-$CD4$^+$ cells alone or together with CD4$^+$YFP$^+$ Treg cells from the spleens of *Foxp3*$^{YFP\text{-}Cre}$ and *SRC2*$^{fl/fl}$/*Foxp3*$^{YFP\text{-}Cre}$ mice (n≥4 per genotype). **C** Representative flow cytometric analysis (left panels) and the relative proliferation (right panel) of responder T cells cultured with varying ratios of splenic YFP$^+$CD4$^+$ Treg cells isolated from indicated mice of 26 weeks old (n=4 per genotype). Data are from three experiments (**A**, **B**, **C**, right panels; presented as mean ± s.d.) or are from one representative of three independent experiments (**A**, **B**, **C**, left panels). *P<0.05; **P<0.01; ns, not significant (two-tailed Students' t-test).

Fig. S5. SRC2 stimulates the expression of *Nr4a2* critical for Treg differentiation.

A Flow cytometric analysis of Foxp3 levels (left two panels) and relative degradation rate (right panel) in Treg differentiated *in vitro* from *Foxp3*$^{YFP\text{-}Cre}$ and *SRC2*$^{fl/fl}$/*Foxp3*$^{YFP\text{-}Cre}$ CD4$^+$ T cells and treated with protein synthesis inhibitor CHX for different times (n=4 per genotype). **B** Schematic representation of RNA-seq analysis groups of indicated genotype of naïve CD4$^+$ cells and CD4$^+$ cells polarized under Treg differentiation conditions for 36 hours (n=3 per genotype per group) (left panel). Expression of Foxp3 after 36 hours Treg differentiation in indicated mice (n=3 per genotype) (middle panel). Right panel is the principal component analysis (PCA) of transcriptomes in four groups shown on left. **C** List of genes known to regulate the Treg differentiation. **D** Representative flow cytometric analysis of Foxp3 (left panel) and the percentage (right panel) of Foxp3$^+$ cells among *SRC2*$^{fl/fl}$/*CD4*^{Cre} CD4$^+$ cells transduced with virus expressing GFP alone (EV) or together with *Foxo1, Irf4* or *Myb* and polarized under Treg conditions for 48 hours (n≥3 per treatment per group). **E** Representative flow cytometric analysis of Foxo1 (left panel) and MFI (right panel) for Foxo1 among indicated genotypes of CD4$^+$ cells polarized under Treg conditions for 24 hours and 48 hours (n≥3 per treatment per group). **F** Representative flow cytometric analysis of IRF4 (left panel) and MFI (right panel) for IRF4 among indicated genotypes of CD4$^+$ cells polarized under Treg conditions for 24 hours and 48 hours (n≥3 per treatment per group). **G** Representative flow cytometric analysis of Foxp3 (left panels) and the percentage (right panel) of Foxp3$^+$ Tregs among *SRC2*$^{fl/fl}$/*CD4*^{Cre} NGFR$^+$CD4$^+$ cells transduced by retrovirus expressing NGFR alone (EV) or together with *Nr4a2* (Nr4a2) and polarized under Treg conditions for 24 hours or 48 hours (n≥4 per treatment per group). **H** Representative flow cytometric analysis

of GFP and NGFR (top two left panels), and Foxp3 (bottom two left panels) in gated GFP^+NGFR^+ cells among *SRC2*$^{fl/fl}$/*CD4*^{Cre} $CD4^+$ T cells transduced with retrovirus expressing NGFR/Nr4a2 together with virus expressing GFP (EV+*Nr4a2*) or together with virus expressing GFP/IRF4 (*Irf4*+*Nr4a2*) and polarized under Treg conditions for 48 hours (n≥3 per treatment per group). Right panel is the summary of the percentage of $Foxp3^+$ Tregs among $GFP^+NGFR^+CD4^+$ cells. **I** Representative flow cytometric analysis of GFP and NGFR (top two left panels), and Foxp3 (bottom two left panels) in gated GFP^+NGFR^+ cells among *SRC2*$^{fl/fl}$/*CD4*^{Cre} $CD4^+$ T cells transduced with retrovirus expressing NGFR/Nr4a2 (*Nr4a2*) or together with virus expressing GFP/Foxo1 (*Foxo1*+*Nr4a2*) and polarized under Treg conditions for 48 hours (n≥3 per treatment per group). Right panel is the summary of the percentage of $Foxp3^+$ Tregs among $GFP^+NGFR^+CD4^+$ cells. Boxed region: cell population of interest. Data are from three experiments (**A, D, E, F, G,** right panels; presented as mean ± s.d.), two experiments (**H, I,** right panels; presented as mean ± s.d.) or are from one representative of three or two independent experiments (**A, D, E, F, G, H, I,** left panels). *P<0.05; ns, not significant (two-tailed Students' *t*-test in **A**, **E**, **F**, **G**, **H**, **I** and one-way ANOVA with Tukey's post-analysis multiple-comparison test in **D**).

Fig. S6. SRC2 recruited by NFAT1 binds to the promoter and activate gene expression of *Nr4a2.*

A SRC2 DNA-binding signals in the *Nr4a2* promoter region, detected by ChIP-seq assay, in indicated $CD4^+$ cells 36 hours post Treg polarization. **B** Transcriptional factor-binding sites predicted by PROMO in the P0-P2 region of *Nr4a2* promoter (similarity >95%). **C** Full-length image of immunoblot assay shown in figure 6**D**. **D** Schematic representation of process of CRISPR/Cas9-mediated deletion of target DNA fragment. **E** Full-length image of immunoblot analysis of the expression of Nr4a2 shown in 6**F**. F Schematic representation of the locations of the six regions on *Nr4a2* promoter covered by P0-P5 primers, and the crNr4a2 region shown in Fig. 6A and the negative control region (crNeg) deleted using CRISPR/Cas9. **G** PCR analysis of the abundance of negative control region on *Nr4a2* promoter in $CD4^+$ cells transduced with retrovirus expressing guiding RNAs, either non targeting control (NTC) or deletion of the negative control region (crNeg) showing in F, and polarized under Treg condition for 40 hours. **H** Immunoblot analysis of Nr4a2 in naïve $CD4^+$ cells or $CD4^+$ T cells transduced with virus expressing NTC and crNeg shown in **F** and polarized under Treg conditions for 40 hours. Number in the bottom of the blots is the relative mean intensity of the band analyzed by histogram, and the right panel is the summary of the relative mean intensity of each band. **I** Representative flow cytometric analysis of Foxp3 (left panels), percentages of $Foxp3^+$ cells (middle panels) and MFI for Foxp3 (right panels) among $GFP^{hi}CD4^+$ cells transduced with virus expressing *NTC, crFoxp3*, *crNr4a2* and *crNeg* guiding RNAs, and polarized under Treg condition for 60 hours (n≥3 per treatment per group). Data are from two (**H**, right panel) or three experiments (**I**, middle and right panels; presented as mean ± s.d.) or are from one representative of three independent experiments (**C**, **E, G**; **H**, **I**, left panels). **P<0.01; **** P<0.0005; ns, not significant (two-tailed Students' *t*-test).

Table S1.

List of primers and guide RNA sequences used in this study.

Figure S1

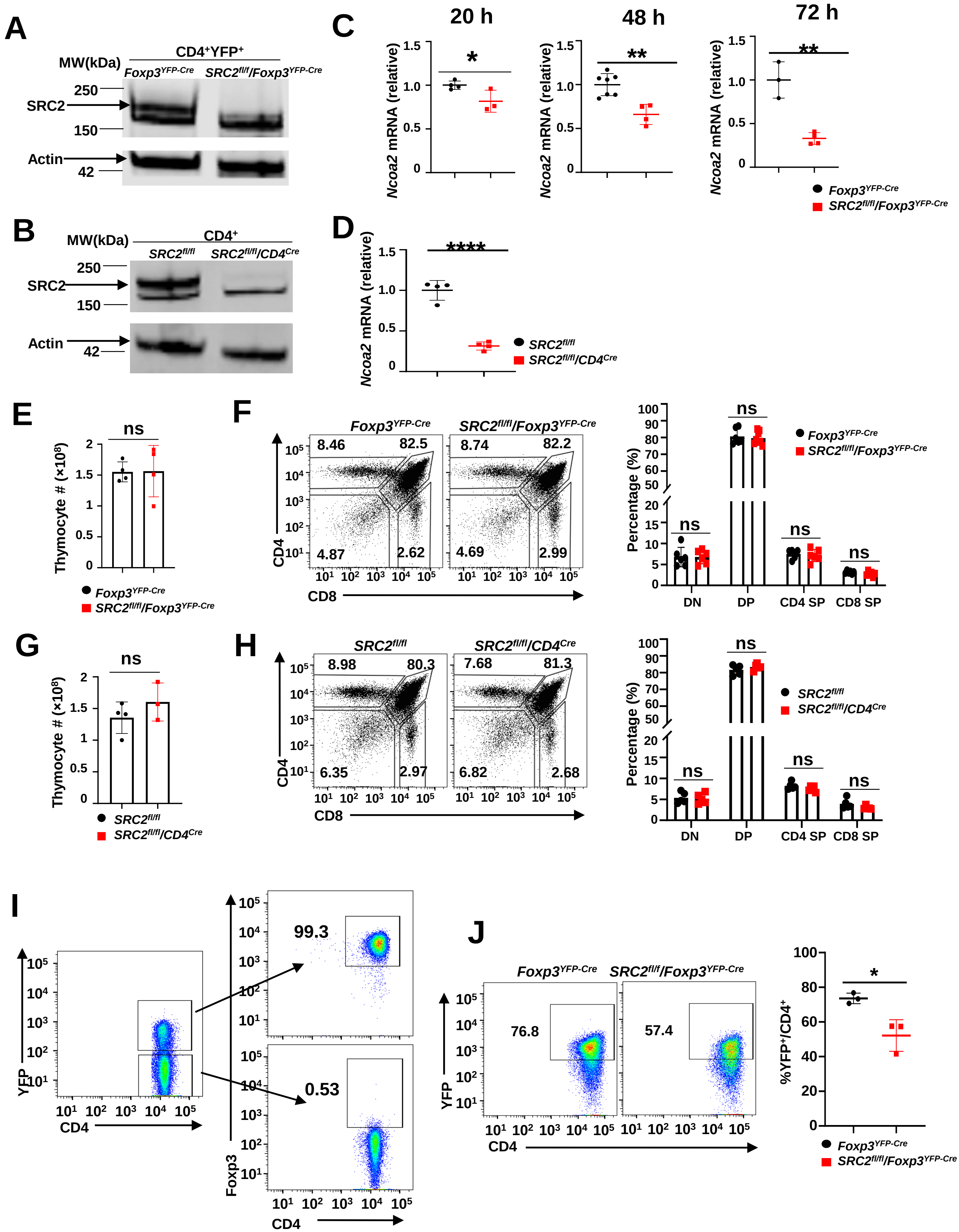
A
MW(kDa)
CD4+YFP+
Foxp3YFP-Cre
SRC2fl/f/Foxp3YFP-Cre
250
SRC2
150
Actin
42
B
MW(kDa)
CD4+
SRC2fl/fl
SRC2fl/fl/CD4Cre
250
SRC2
150
Actin
42
C
20 h
48 h
72 h
Ncoa2 mRNA (relative)
*
**
**
Foxp3YFP-Cre
SRC2fl/fl/Foxp3YFP-Cre
D
Ncoa2 mRNA (relative)
****
SRC2fl/fl
SRC2fl/fl/CD4Cre
E
ns
Thymocyte # (×10⁸)
Foxp3YFP-Cre
SRC2fl/fl/Foxp3YFP-Cre
F
Foxp3YFP-Cre
SRC2fl/fl/Foxp3YFP-Cre
8.46
82.5
4.87
2.62
8.74
82.2
4.69
2.99
CD4
CD8
Percentage (%)
ns
DN
DP
CD4 SP
CD8 SP
G
ns
Thymocyte # (×10⁸)
SRC2fl/fl
SRC2fl/fl/CD4Cre
H
SRC2fl/fl
SRC2fl/fl/CD4Cre
8.98
80.3
6.35
2.97
7.68
81.3
6.82
2.68
CD4
CD8
Percentage (%)
ns
DN
DP
CD4 SP
CD8 SP
I
YFP
CD4
99.3
0.53
Foxp3
CD4
J
Foxp3YFP-Cre
SRC2fl/fl/Foxp3YFP-Cre
76.8
57.4
YFP
CD4
%YFP+/CD4+
*
Foxp3YFP-Cre
SRC2fl/fl/Foxp3YFP-Cre

Figure S1 -continued

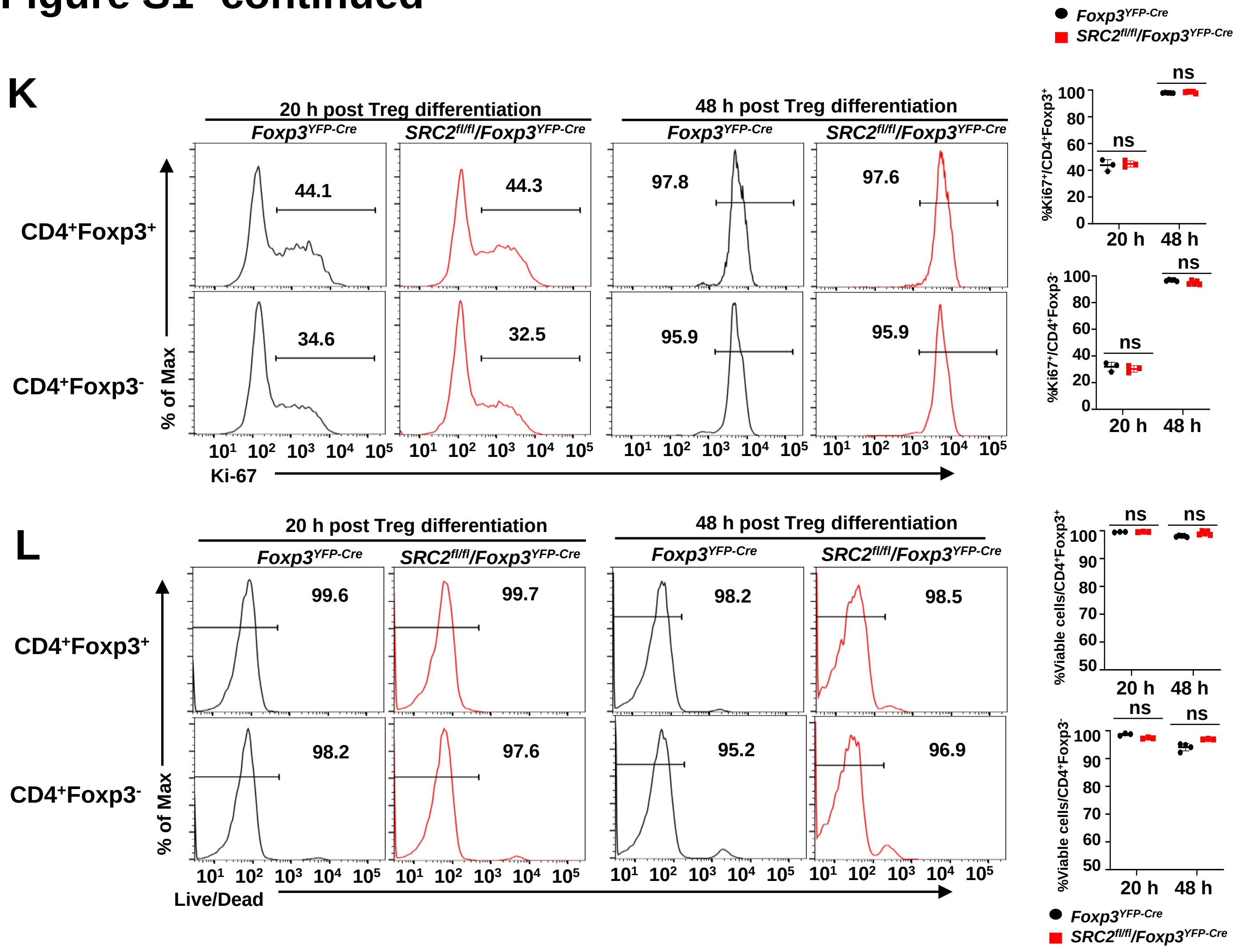

Figure S2

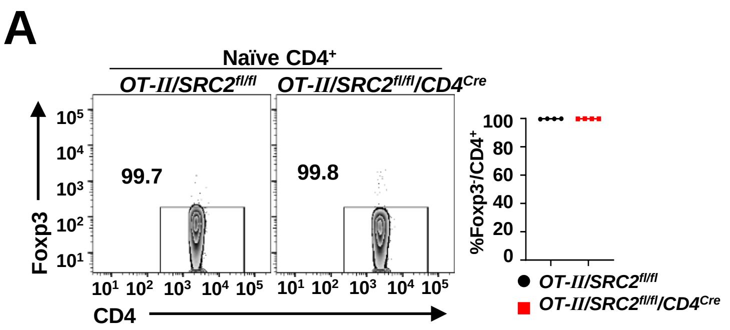
A
Naïve CD4+
OT-II/SRC2fl/fl
OT-II/SRC2fl/fl/CD4Cre
Foxp3
CD4
99.7
99.8
%Foxp3-/CD4+
OT-II/SRC2fl/fl
OT-II/SRC2fl/fl/CD4Cre

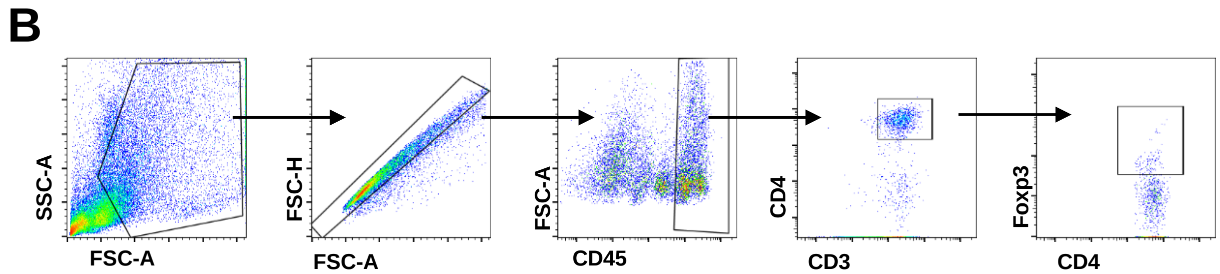
B
SSC-A
FSC-A
FSC-H
FSC-A
FSC-A
CD45
CD4
CD3
Foxp3
CD4

Figure S3

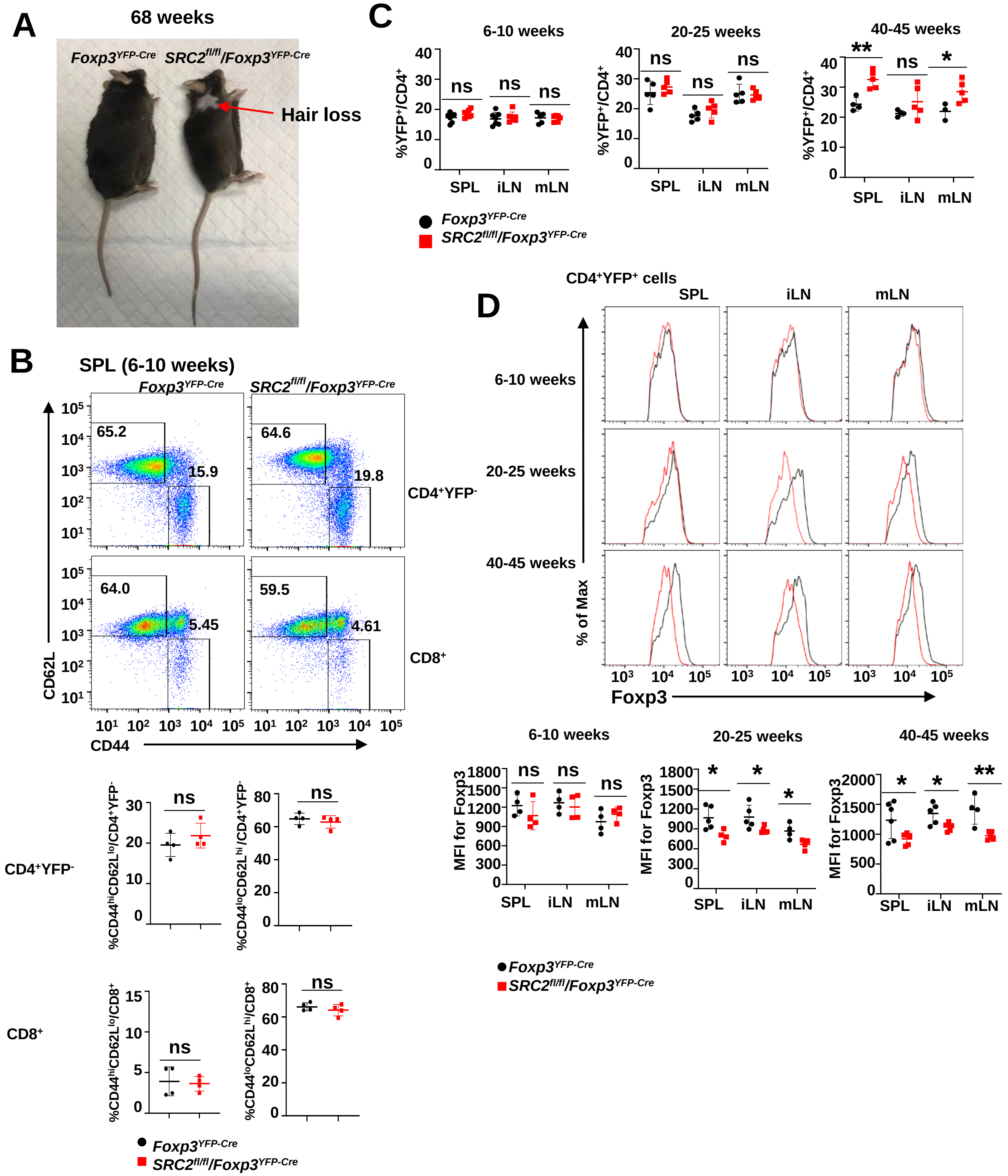

A
68 weeks
Foxp3YFP-Cre
SRC2fl/fl/Foxp3YFP-Cre
Hair loss
C
6-10 weeks
20-25 weeks
40-45 weeks
%YFP+/CD4+
ns
**
*
SPL
iLN
mLN
Foxp3YFP-Cre
SRC2fl/fl/Foxp3YFP-Cre
B
SPL (6-10 weeks)
65.2
15.9
64.6
19.8
64.0
5.45
59.5
4.61
CD4+YFP-
CD8+
CD62L
CD44
%CD44hiCD62Llo/CD4+YFP-
%CD44loCD62Lhi/CD4+YFP-
%CD44hiCD62Llo/CD8+
%CD44loCD62Lhi/CD8+
D
CD4+YFP+ cells
% of Max
Foxp3
MFI for Foxp3

Figure S3 -continued

E

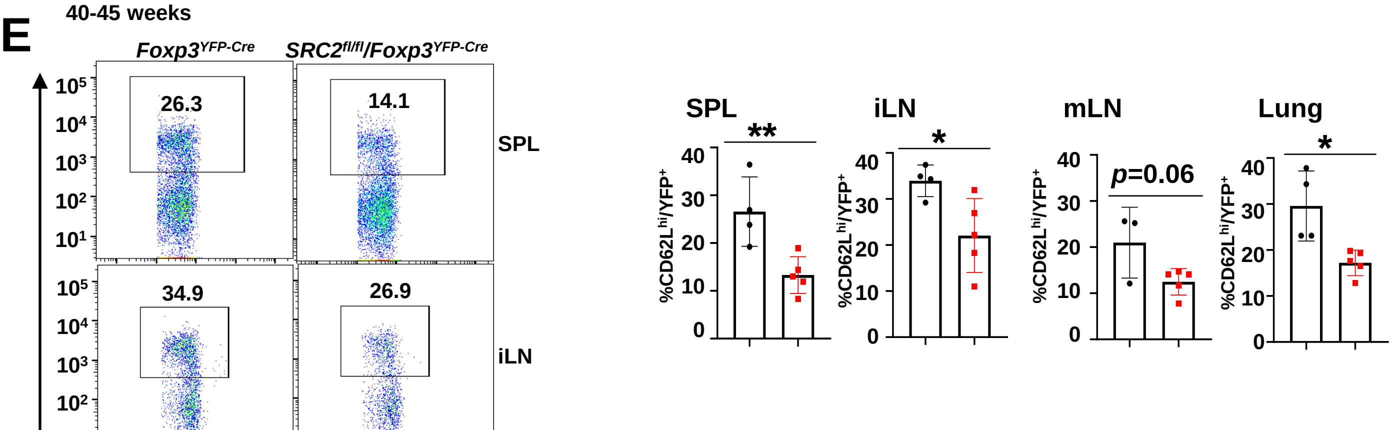

F

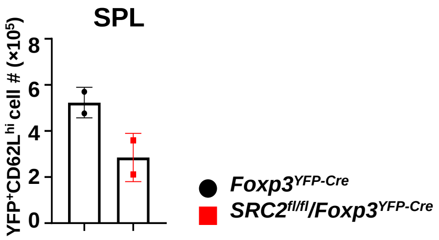

Figure S4

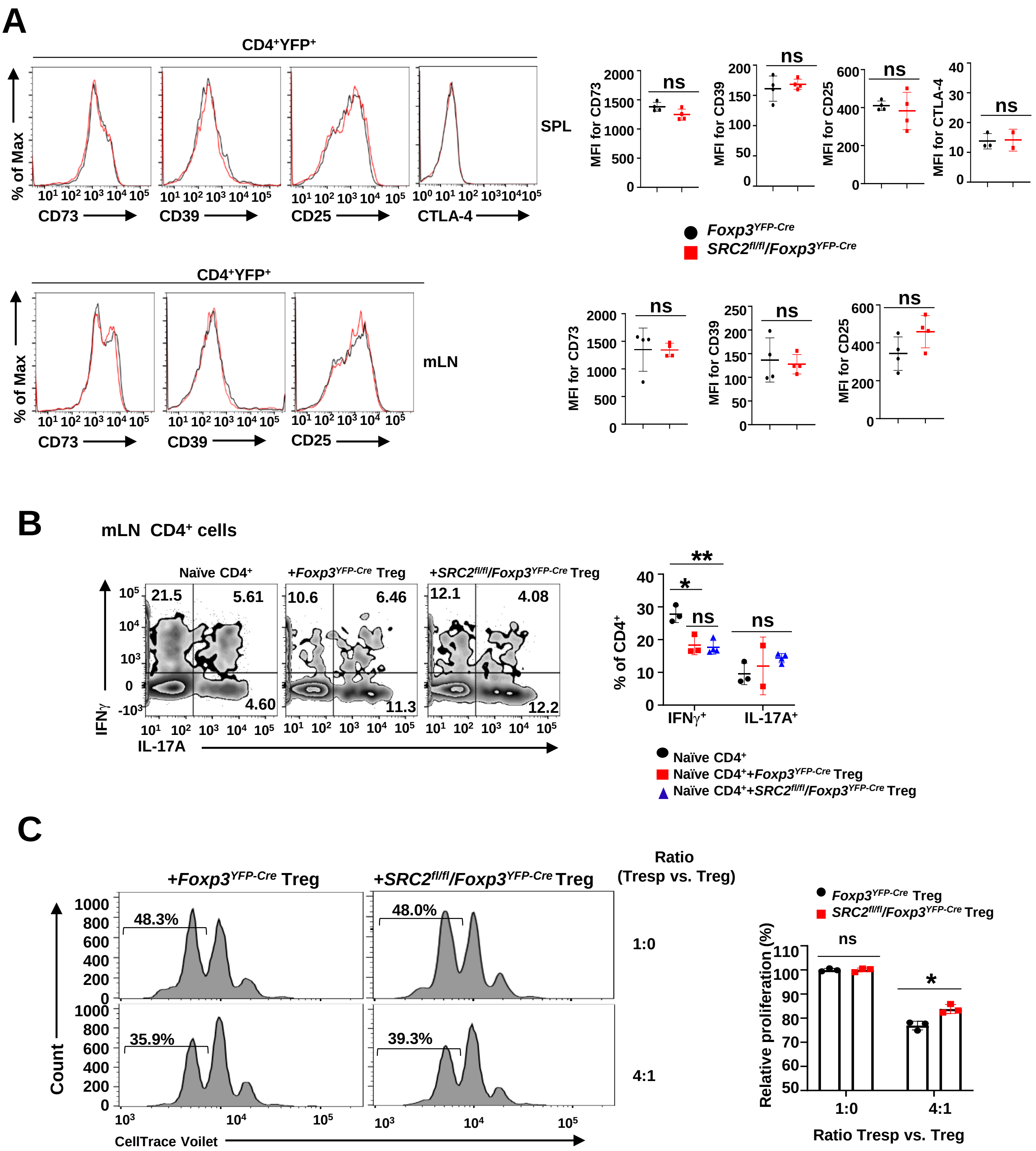
A
CD4+YFP+
SPL
% of Max
CD73
CD39
CD25
CTLA-4
MFI for CD73
MFI for CD39
MFI for CD25
MFI for CTLA-4
ns
Foxp3YFP-Cre
SRC2fl/fl/Foxp3YFP-Cre
mLN
B
mLN CD4+ cells
Naïve CD4+
+Foxp3YFP-Cre Treg
+SRC2fl/fl/Foxp3YFP-Cre Treg
21.5
5.61
4.60
10.6
6.46
11.3
12.1
4.08
12.2
IFNγ
IL-17A
% of CD4+
IFNγ+
IL-17A+
**
*
Naïve CD4+
Naïve CD4++Foxp3YFP-Cre Treg
Naïve CD4++SRC2fl/fl/Foxp3YFP-Cre Treg
C
Ratio
(Tresp vs. Treg)
48.3%
48.0%
35.9%
39.3%
1:0
4:1
Count
CellTrace Voilet
Foxp3YFP-Cre Treg
SRC2fl/fl/Foxp3YFP-Cre Treg
Relative proliferation (%)
Ratio Tresp vs. Treg

Figure S5

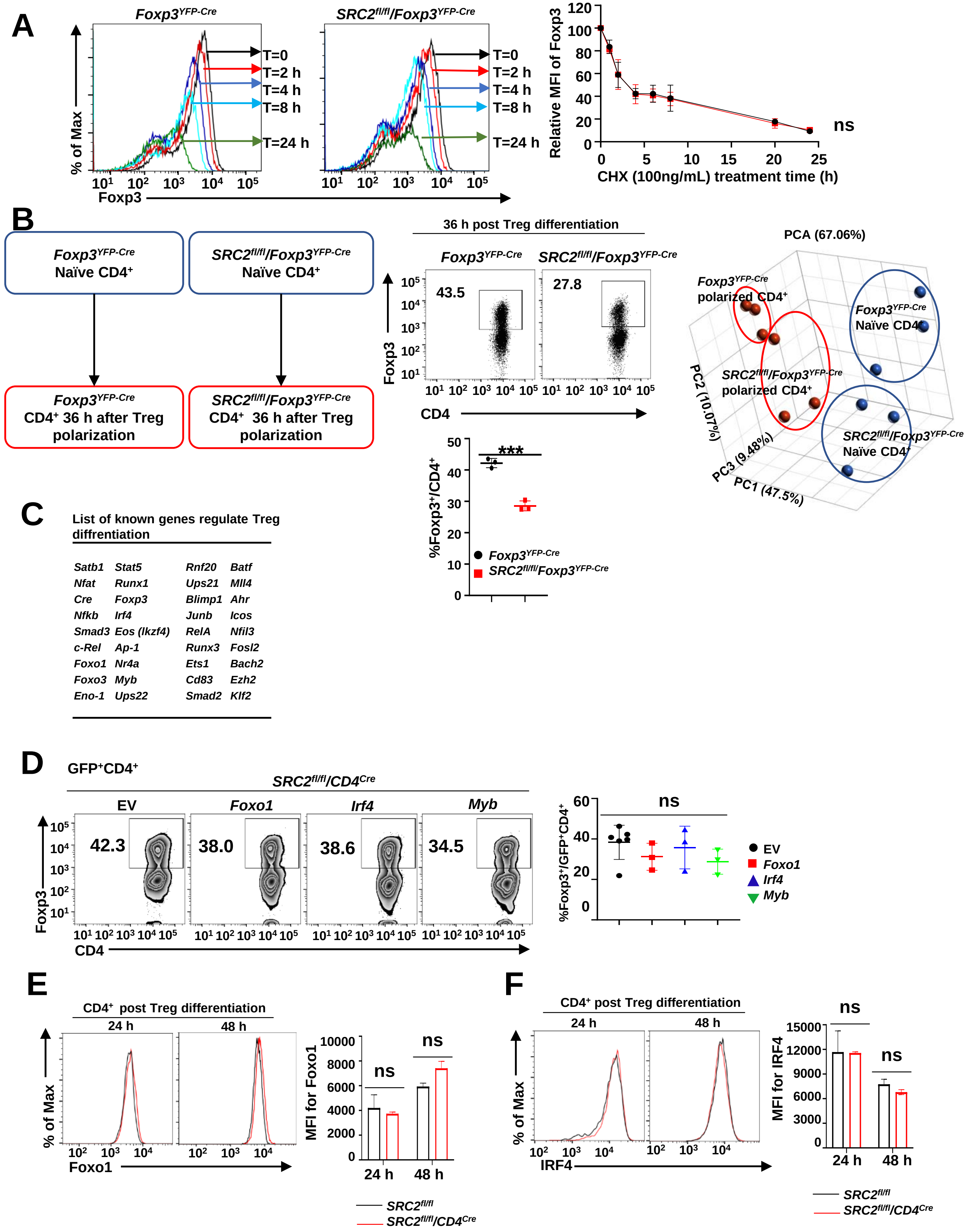
A
Foxp3YFP-Cre
SRC2fl/fl/Foxp3YFP-Cre
T=0
T=2 h
T=4 h
T=8 h
T=24 h
% of Max
Foxp3
Relative MFI of Foxp3
CHX (100ng/mL) treatment time (h)
ns
B
Foxp3YFP-Cre Naïve CD4+
SRC2fl/fl/Foxp3YFP-Cre Naïve CD4+
Foxp3YFP-Cre CD4+ 36 h after Treg polarization
SRC2fl/fl/Foxp3YFP-Cre CD4+ 36 h after Treg polarization
36 h post Treg differentiation
43.5
27.8
CD4
Foxp3
***
%Foxp3+/CD4+
PCA (67.06%)
Foxp3YFP-Cre polarized CD4+
SRC2fl/fl/Foxp3YFP-Cre polarized CD4+
Foxp3YFP-Cre Naïve CD4+
SRC2fl/fl/Foxp3YFP-Cre Naïve CD4+
PC2 (10.07%)
PC3 (9.48%)
PC1 (47.5%)
C
List of known genes regulate Treg diffrentiation
Satb1 Stat5 Rnf20 Batf
Nfat Runx1 Ups21 Mll4
Cre Foxp3 Blimp1 Ahr
Nfkb Irf4 Junb Icos
Smad3 Eos (Ikzf4) RelA Nfil3
c-Rel Ap-1 Runx3 Fosl2
Foxo1 Nr4a Ets1 Bach2
Foxo3 Myb Cd83 Ezh2
Eno-1 Ups22 Smad2 Klf2
D
GFP+CD4+
SRC2fl/fl/CD4Cre
EV
Foxo1
Irf4
Myb
42.3
38.0
38.6
34.5
Foxp3
CD4
ns
%Foxp3+/GFP+CD4+
E
CD4+ post Treg differentiation
24 h
48 h
% of Max
Foxo1
MFI for Foxo1
ns
SRC2fl/fl
SRC2fl/fl/CD4Cre
F
CD4+ post Treg differentiation
24 h
48 h
% of Max
IRF4
MFI for IRF4
ns
SRC2fl/fl
SRC2fl/fl/CD4Cre

Figure S5 -continued

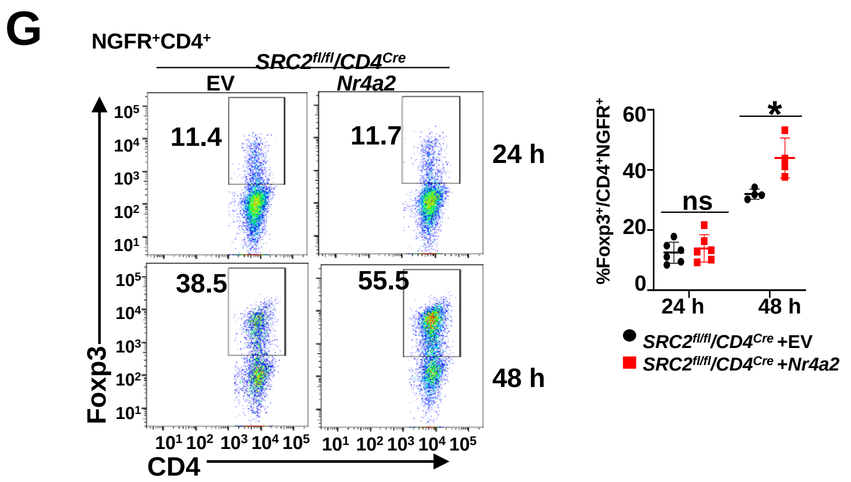

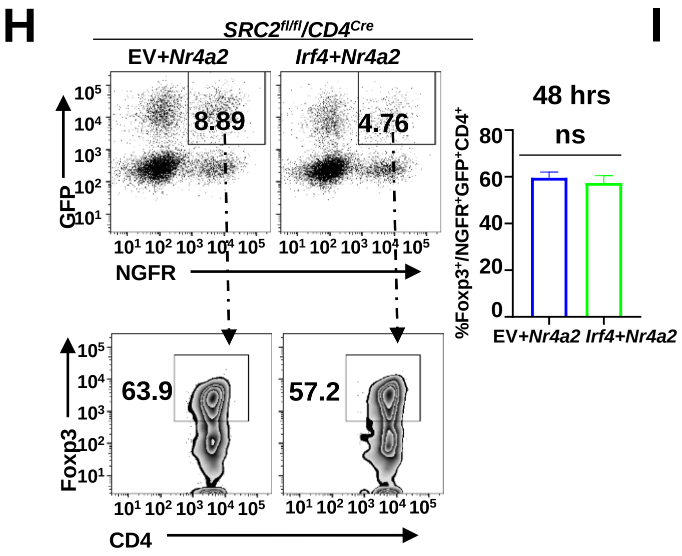

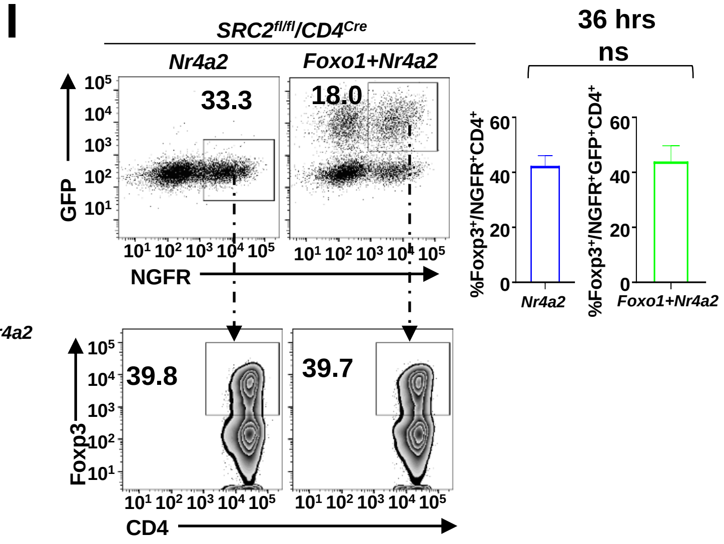

Figure S6

A

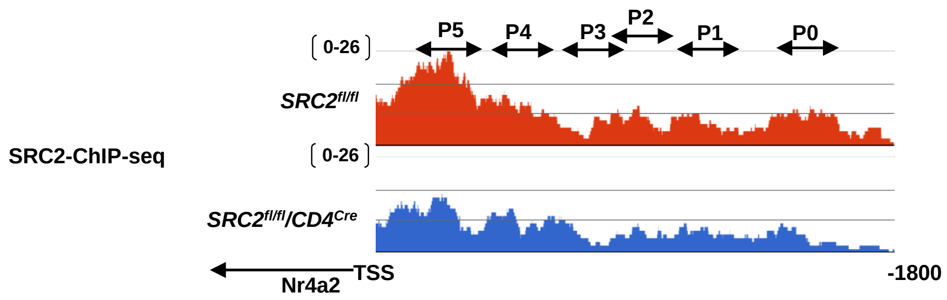

B

T-cell activation related transcriptional factors predicted by PROMO in the region of P0-P2 (similarity >95%)

STAT4
c-Jun
NFAT3
NFAT1
NFAT2
STAT6

C

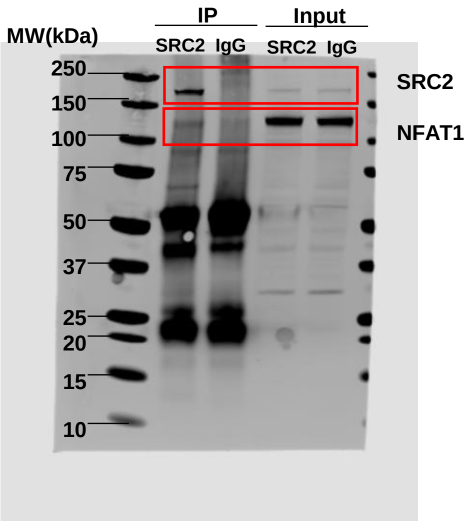

E

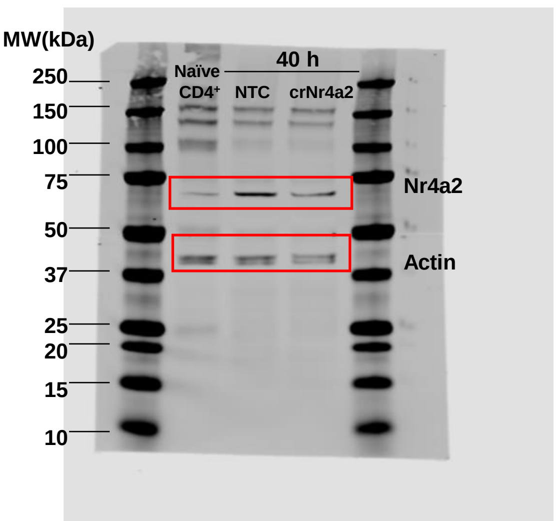

D

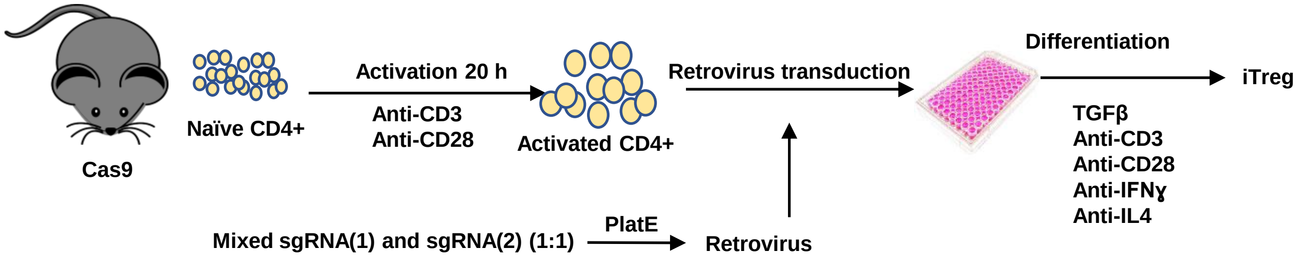

Figure S6 -continued

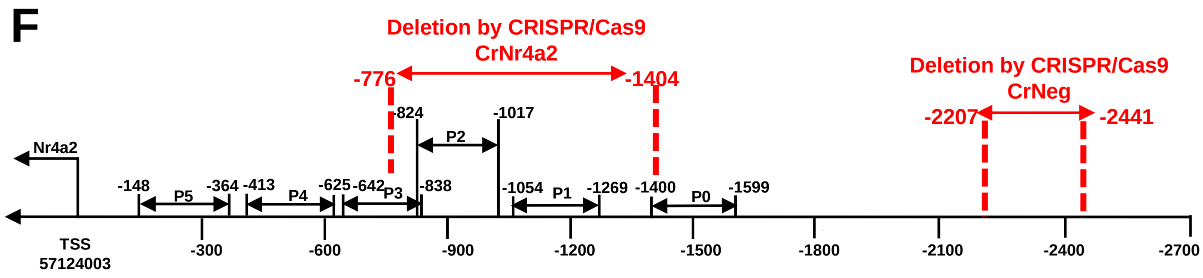

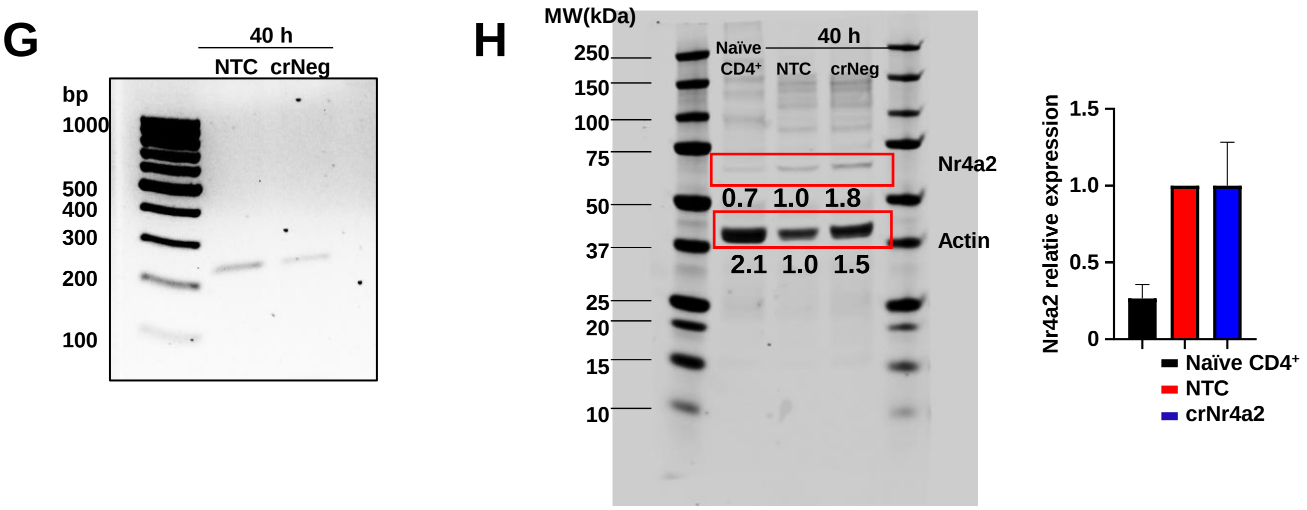

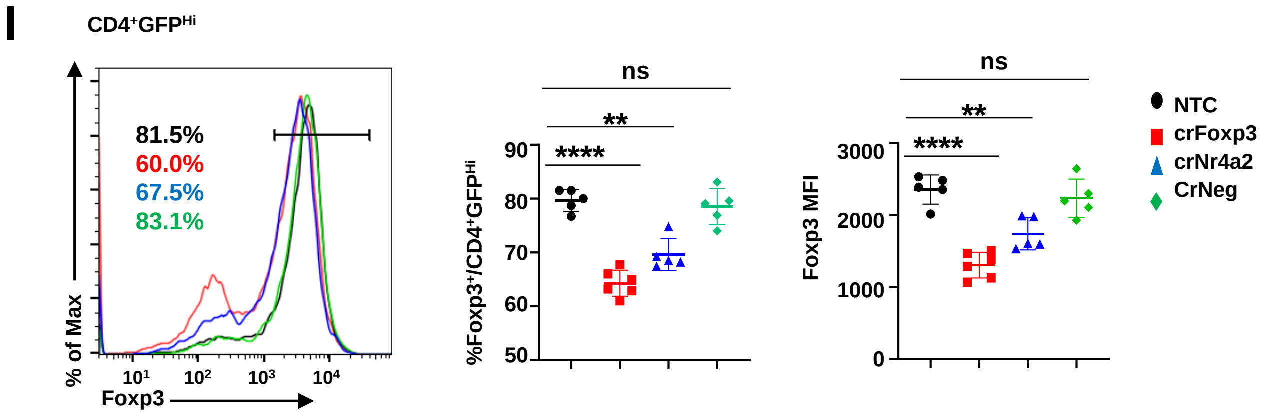

Table S1

qPCR	Gene	Forward	Reverse
	Foxp3	CCCATCCCCAGGAGTCTTG	ACCATGACTAGGGGCACTGTA
	Ncoa2	GACAGCGGCCAAATTACACC	ATAAGCGGCTGGCGATTCTG
	Foxo1	CCCAGGCCGGAGTTTAACC	GTTGCTCATAAAGTCGGTGCT
	Myb	AGACCCCGACACAGCATCTA	CAGCAGCCCATCGTAGTCAT
	Irf4	TCCGACAGTGGTTGATCGAC	CCTCACGATTGTAGTCCTGCTT
	Nr4a2	GTGTTCAGGCGCAGTATGG	TGGCAGTAATTTCAGTGTTGGT
	Stat5a	CGCCAGATGCAAGTGTTGTAT	TCCTGGGGATTATCCAAGTCAAT
	Actin	GGGAAATCGTGCGTGACAT	GTCAGGCAGCTCGTAGCTCTT

ChIP-qPCR	Binding site	Forward	Reverse
	P0	GTCTAGGAAGGTCAGTGAGA	ATGCTGGTAGCTGTGTTC
	P1	AGAGTCCAGCGAAGACATA	CCTCAGGCATAGGAACTCTA
	P2	GAGATGACCACAAGAGTTGG	CCCGTAGATGAATGAAGATCAG
	P3	TTCCAGCAGAGTAGCTGAT	CTTCCAAGTCTGGTCCTTTG
	P4	CATCCTGAAGGCCACAAA	CTCCACTACAAAGTCCAGTG
	P5	GAACTTGCAGAGGAGTTGAG	CAGCCGGCGCTATAAATAA

CRISPR sgRNA		Sequence
	NTC (1)	GCGAGGTATTCGGCTCCGCG
	NTC (2)	GCTTTCACGGAGGTTCGACG
	crNr4a2 (1)	GCATGGAAAGGAAACAACGG
	crNr4a2 (2)	GATGAATAAGACACGCGTCA
	crFoxp3 (1)	GCACCGTCTGGGGCCCGACT
	crFoxp3 (2)	GGTTGTCCAGTGGACGCACT
	crNeg (1)	GGGTATGTTTAATATTTACT
	crNeg (2)	TTGTGCATTCATCATAGGAG

PCR		Forward	Reverse
	**NFAT1/SRC2 binding region on Nr4a2 promoter**	CACCGGCATGGAAAGGAAACAACGG	AAACTGACGCGTGTCTTATTCATCC
	**Negative control region on Nr4a2 promoter**	CACCGGGGTATGTTTAATATTTACT	CACCGTTGTGCATTCATCATAGGAG
